# The impacts of a global pandemic on the efficacy and stability of contemporary wildlife conservation: South Africa as a case study

**DOI:** 10.1007/s13280-022-01814-z

**Published:** 2022-12-30

**Authors:** David A. Ehlers Smith, Yvette C. Ehlers Smith, Harriet T. Davies-Mostert, Lindy J. Thompson, Daniel M. Parker, Deon de Villiers, Dean Ricketts, Brent Coverdale, Peter J. Roberts, Christopher Kelly, Duncan N. Macfadyen, Nomthandazo S. Manqele, R. John Power, Colleen T. Downs

**Affiliations:** 1 Centre for Functional Biodiversity, School of Life Sciences, University of KwaZulu-Natal, Private Bag X01, Scottsville, Pietermaritzburg, 3209 South Africa; 2 Ezemvelo KwaZulu-Natal Wildlife, Queen Elizabeth Park, Peter Brown Drive, Montrose, Pietermaritzburg, 3201 South Africa; 3 Endangered Wildlife Trust, Midrand, 1685 South Africa; 4 Department of Zoology and Entomology, Mammal Research Institute, University of Pretoria, Private Bag X20, Hatfield, Pretoria, 0028 South Africa; 5 Conserve Global, London, W1G 8TB UK; 6 School of Biology and Environmental Sciences, University of Mpumalanga, Mbombela, 1200 South Africa; 7 Wildlife and Reserve Management Research Group, Department of Zoology and Entomology, Rhodes University, Makhanda, 6140 South Africa; 8 Compliance and Enforcement, Department of Economic Development, Environmental Affairs and Tourism, Eastern Cape, Bisho, South Africa; 9 Department of Economic Development, Environmental Affairs and Tourism, Eastern Cape, Bisho, South Africa; 10 Wildlife ACT Fund Trust, Gardens, Cape Town, 8001 South Africa; 11 Department of Research and Conservation, Oppenheimer Generations, 6 St Andrews Road, Parktown, Johannesburg, 2139 South Africa; 12 Department of Economic Development, Environment, Conservation & Tourism, North-West Provincial Government, NWDC Building, Mmabatho, 2750 South Africa

**Keywords:** Compliance, Hunting, Illegal hunting, Land invasions, Poaching, Wildlife tourism

## Abstract

Conservationists speculated on potential benefits to wildlife of lockdown restrictions because of the COVID-19 pandemic but voiced concern that restrictions impeded nature conservation. We assessed the effects of lockdown restrictions on biodiversity conservation in South Africa, a biodiverse country with economic inequality and reliance on wildlife resources. We solicited expert opinion using the IUCN’s Threats Classification Scheme to structure a questionnaire and illustrated responses with individual case studies from government parastatal and non-governmental conservation organisations. The most highly reported threats were biological resource use, residential/commercial developments, invasive species, and human intrusions. The trends reported by 90 survey respondents were supported by case studies using environmental compliance data from parastatal conservation organisations. Lack of tourism revenue and funding were cited as hindrances to conservation. Mechanisms to prevent environmental degradation in the face of global emergencies must be implemented and ‘ring-fenced’ to ensure conservation is not a casualty during future global crises.

**Supplementary Information:**

The online version contains supplementary material available at 10.1007/s13280-022-01814-z.

## Introduction

The anthropogenic destruction of wild spaces is driving a global mass extinction of species (IPBES 2019). The increasing proximity and frequency of contact between people, livestock, and wild animals may increase the risk of zoonotic disease transmission and allow mutations of novel pathogens with subsequent human–human transmission (Chomel et al. 2007; Smith and Wang 2013; Wynne and Wang 2013). Reducing human encroachment into intact ecosystems and the consumptive use of wildlife may in turn reduce the risk of zoonotic disease transmission (Wolfe et al. 2005; Ahmad et al. 2020). However, the rapid expansion of the global human population and its associated resource requirements mean a reduction in wildlife consumption is unlikely (McLaughlin 2011). Human population growth and in some regions, our reliance on biological resources, are often greatest in poverty-stricken regions, thus complicating conservation issues with ethical obligations to ensure vulnerable human populations have access to food, shelter and other basic needs (Kiesecker et al. 2019).

Previous zoonotic disease outbreaks, such as the intercontinental spread of Severe Acute Respiratory Syndrome (SARS) in 2004, reaffirmed that the threats from zoonotic diseases are constantly and increasingly present (Taylor et al. 2001) and highlighted the lack of preparedness of governments to respond to such events (Ostfeld and Holt 2004). In December 2019, a novel pathogen called Severe Acute Respiratory Syndrome Coronavirus 2 (SARS-CoV-2, which causes the human disease coronavirus 19, COVID-19) emerged in Wuhan, China. Present evidence indicates the virus was zoonotic in origin (Wong et al. 2020).

Since the initial outbreak, COVID-19 has spread to virtually every country and territory on Earth, and was declared a global pandemic by the World Health Organization (WHO) on 11 March 2020. As of May 2022, over 515 million cases of COVID-19 have been reported globally, with 6.3 million fatalities (World Health Organization 2022). Governments have responded by implementing restrictions (called “lockdown” measures), on the movements of their citizens, with varying degrees of success in containing the spread of the virus (Ajide et al. 2020; Meunier 2020). Subsequent waves of infection, driven by various mutations in the virus that have increased its transmissibility, resulted in continued lockdown measures, which forced citizens and governments to adapt to drastic curtailments of freedom (Aragona et al. 2020; Meunier 2020).

The COVID-19 pandemic, and conservation practitioners’ responses, may be considered in four broad contexts. Firstly, conservationists speculated on the potential benefits to wildlife of lockdown restrictions, which curtailed people’s movements; the ‘anthropause’ (c.f. Rutz et al. 2020; Ferreira et al. 2021). This gave natural environments temporary respite from human disturbance and resource extraction (Bang and Khadakkar 2020; Corlett et al. 2020; Montgomery et al. 2020; Mostafa and Gamil Gamal 2020; Neupane 2020; Rutz et al. 2020). Secondly, conservationists voiced concern that lockdown restrictions impeded their ability to practice nature conservation. Travel restrictions, reductions and retractions of funding, and impediments to training, research, monitoring, and conservation law enforcement were cited as hindrances (Buxton et al. 2020; Corlett et al. 2020; Daly 2020; Evans et al. 2020; Lindsey et al. 2020; McLeery et al. 2020; Swing et al. 2020; but see Ferreira et al. 2021). Thirdly, conservation biologists recognised the inherent potential for a ‘global human confinement experiment’ to investigate the positive and negative influences of human presence in (and absence from) wild spaces (Cooke et al. 2021; Manenti et al. 2020; Montgomery et al. 2020; Rutz et al. 2020). Lastly, the potential for an evaluation of what may be improved in conservation practice, as highlighted by the pandemic, and how to proceed in a post-COVID-19 era has been widely discussed (Bang and Khadakkar 2020; Buxton et al. 2020; Cawthorn et al. 2020; Cooke et al. 2020; Evans et al. 2020; Kavousi et al. 2020; Sandbrook et al. 2020).

In South Africa, the initial lockdown measures implemented by the national government were some of the most restrictive globally (Table 1). A horizon scan of conservation threats in South Africa highlighted that environmental protection is often side-lined in emergency situations, favouring quick responses to human needs (Seymour et al. 2019). This situation warrants investigation into how responses to the pandemic affected biodiversity conservation in one of the 17 most mega-biodiverse countries on Earth (WCMC 1992).

**Table 1:** Lockdown restrictions imposed by the South African government during between the first and the second waves of infection of the COVID-19 pandemic (www.gov.za)

Lockdown	Timeframe	Curfew	Conditions
Level 5	Mar 26–May 01 2020	Continual; all citizens must remain at home except for purchase of provisions and medical emergencies	Only essential services operating: health workers, pharmacy and laboratory personnel, emergency personnel; security services (police officers, military personnel, and private security; people regarded as necessary to the basic functioning of the economy (supermarkets, transportation and logistical services, petrol stations, banks, essential financial and payment services); and those working in industries that cannot be economically shut down (e.g. mines and steel mills); liquor and alcohol sales prohibited
Level 4	May 02–Jun 01 2020	20:00–05:00; citizens allowed out from 06:00–09:00 within a 5 km radius of home	Local, provincial and international travel banned, services opened for food, cleaning, protective, baby care, stationery; winter clothing, bedding, heating; medical supplies; fuel, coal, wood, gas; hardware supplies for emergency home repairs and essential services by qualified tradespersons; components for vehicles for essential workers; chemicals, packaging, and supply of Level 4 products; liquor and alcohol sales prohibited
Level 3	Jun 02–Aug 17 2020	21:00–04:00	Local, provincial and international travel banned, places closed to public, all businesses may operate except liquor and tobacco retailers; short-term home rental for leisure purposes; passenger ships for leisure purposes; and entertainment activities; reserves open for self-drive reserves but overnight guests prohibited
Level 2	Aug 18–Sep 21 2020	22:00–04:00	Inter-provincial travel ban lifted, international travel limitations, gathering restrictions eased and gyms open, overnight accommodation permitted, reserves open for overnight guests
Level 1	September 22–December 28 2020	0:00–04:00	All sectors open; gatherings limited to 250 people at indoor events and 500 people at outdoor events; physical exercise, recreation and entertainment venues allowed to operate at 50% capacity

South Africa's economy relies heavily on the wildlife tourism sector (Taylor et al. 2016). Yet, government spending on environmental protection is below the global average and accounted for 0.7% of the annual budget in 2013/14 (Statistics SA 2015). In addition, South Africa has pronounced economic inequality, and its large, rural, historically-disadvantaged human population is heavily reliant on natural resources for daily survival, and often excluded from the economic benefits of ecotourism (Shackleton et al. 2000; Wynburg 2002; De Villiers 2021).

During lockdown restrictions, wildlife management was listed as an essential service by the South African government, and so we believe there is a need to assess the efficacy during this crisis period of all stakeholders involved in conservation activities. We aimed to assess the effects of lockdown restrictions on conservation practitioners’ ability to conduct effective conservation activities in South Africa, a relatively biodiverse country with extreme economic inequality, and widespread reliance on wildlife resources, by involving conservation agencies, practitioners, and conservation biologists. We aimed to solicit expert opinions and experiences via a structured, in-depth questionnaire, and illustrate and expand upon trends in experiences and responses using specific, explicit case studies. These two approaches were selected for their complementary, additive benefits when presented in conjunction, as questionnaires allow for standardised interrogation of multiple issues and may produce high-quality quantitative data (White et al. 2005), while case studies allow for more in-depth exploration of specific issues highlighted (Crowe et al. 2011). We hypothesised that lockdown during the COVID-19 pandemic could affect nature conservation and the wildlife economy sector in South Africa, with nuanced trends across the various lockdown stages (sensu Gibbons et al. 2022). We predicted that (1) reduced tourism and tourism revenue (including that from hunting) would be detrimental to wildlife conservation, given that this underpins conservation in many South African landscapes, and many conservation non-governmental organisations (NGOs) that provide support to conservation efforts are funded by tourism revenue (c.f. Taylor et al. 2019); (2) conservation research and safeguards, including the maintenance and allocation of funds to conduct practical conservation interventions would be negatively impacted by reduced government funding during the pandemic, and by restrictions on movements and access to natural areas, and (3) the impact of a closed economy on poor communities would lead to an increase in biological resource use, including an increase in poaching.

## Materials and Methods

### Questionnaire

On 30 November 2020, 14 conservation practitioners (the authors) from across South Africa met virtually to discuss quantifying the effects of the COVID-19 pandemic, and the various levels of lockdown restrictions, on biodiversity conservation in the country. These people were from a range of academic, governmental, and non-governmental organisations, with expertise in environmental law enforcement, nature conservation, wildlife research, protected area management, and fund-raising. During the meeting, issues pertaining to the effects of the pandemic on biodiversity conservation were presented from first-hand (direct) and second-hand (indirect) accounts, and a framework to rigorously interrogate and quantify the reported effects was developed.

The International Union for the Conservation of Nature’s (IUCN) Threats Classification Scheme v3.2^1^ was adopted as a relevant and established framework upon which to develop a bilingual (English and isiZulu) semi-structured questionnaire on the effects of the pandemic on 12 threat classifications (Table 2). Within each of the 12 classification categories are subcategories, which were summarised for simplicity and ease of use (Supplementary Information S1). Eleven IUCN Threat Categories were retained; the geological event (e.g., earthquakes, floods, etc.) was omitted as irrelevant during the timeframe under investigation (Supplementary Information S1). Issues and experiences of participants could be inserted into the classification framework, whereby each issue pertained to a threat. The responses therein were guided by the framework’s threat impacts in terms of timing, scope, and severity (Supplementary Information S1). The timing options were customised to be relevant to the lockdown timeframes, as specified by the South African government (Table 1), during the period between the first and second waves of COVID-19 infections in South Africa (26 March to 28 December 2020).

**Table 2:** The IUCN’s Threats Classification Scheme was adopted for interrogating the effects of the COVID-19 pandemic and the associated lockdown restrictions on biodiversity conservation in South Africa during 2020

Categories	Levels										
Threats	Residential and commercial developments	Agriculture and aquaculture	Energy production and mining	Transportation and service corridors	Biological resource use	Human intrusion and disturbance	Natural system modification	Invasive species, genes and diseases	Pollution	Climate change and severe weather	Other options
Timing	Short-term (duration of State of Disaster declaration)	Medium-term (3 – 5 years)	Long-term (> 5 years)								
Scope	Affects the minority of the population/ community/ habitat/ ecosystem, etc. (< 50%)	Affects the majority of the population/ community/ habitat/ ecosystem, etc. (50–90%)	Affects the whole population/ community/ habitat/ ecosystem, etc. (> 90%)								
Severity	Threat reduced	Threat increased	Threat stable	Impact on threat suspected but direction unknown	New threat						

Each threat may be considered with any of the options within the timing, scope and severity categories, as relevant and perceived

The 14 authors reached out to various conservation, academic and professional networks to advertise the questionnaire on their mailing lists to elicit respondents. Respondents were asked to describe their area of expertise and the conservation unit (species/community/habitat/ecosystem/locality/bioregion) under discussion for each threat, and they were able to omit any sections for which they could provide no relevant information. Additional unstructured space was provided for respondents to support their responses qualitatively. The questionnaire was produced using the Microsoft Forms© online form generator.^2^ We obtained approval from the University of KwaZulu-Natal’s Humanities Research Ethics Committee before distributing the questionnaire (ethics permit no. HSSREC/00002373/2021).

Respondents were made aware that the questionnaire was optional and not anonymous for two reasons: (1) follow-up data would be requested from certain individuals to quantify their responses, and (2) information pertaining to organisations or sectors would need to be correlated among respondents. Each member of our research team disseminated the questionnaire through their networks (in addition to completing it themselves), targeting academia, government, and non-governmental institutions, including wildlife and zoological associations, conservancies, conservation compliance and law enforcement agencies, and protected area networks.

### Analyses of questionnaire data

Respondents’ areas of expertise were classified into the following levels: Biome; District; National; Protected Areas; Provincial; Regional; and Species. A threat index was devised to calculate the severity of each threat based on the response to each question (Table 3). The sum of each score per question was then pooled per threat to create an overall ranking of threats as perceived by the questionnaire respondents. Likert rankings of participants’ responses were collated and interpreted for all threats, using the ‘*hh*’ package (Heiberger 2022) in R version 4.0.2 (R Core Team 2021).

**Table 3:** Threat categories assigned to the response to each question of the IUCN’s Threats Classification Scheme, adopted for interrogating the effects of the COVID-19 pandemic and the associated lockdown restrictions on biodiversity conservation in South Africa during 2020

Question posed	Threat score
Relevant threat: yes	1
Relevant threat: no	0
Impact yes	2
Impact likely	1
Impact unsure	0
Impact no	− 1
Threat increased	2
New threat	1
Threat stable	0
Threat direction unknown	0
Threat reduced	− 1
Duration of threat long-term	3
Duration of threat medium-term	2
Duration of threat short-term	1
Duration of threat unknown	0
Scope affects entire entity	3
Scope affects majority of entity	2
Scope affects minority of entity	1
Scope unknown	0

Threat categories were applicable to all questions under investigation

### Eastern Cape Province environmental compliance and enforcement unit-case studies

To contextualise the experience of lockdown restrictions on environmental compliance by the government and the enforcement of environmental laws, our survey respondents from the Eastern Cape Province’s Environmental Compliance and Enforcement Unit provided statistics for three regions, namely the Chris Hani, Joe Gqabi and Amathole Districts, in which reporting rates of illegal dog (*Canis lupus familiaris*) hunting incidents were provided for 2019 and 2020, allowing the comparison of frequencies pre- and peri-lockdown. Hunting incidents described were related to illegal poaching activities with the use of dogs to catch and kill wild mammals and birds. We provide a further case study detailing the reports of land invasions into state farmland containing natural forests, as detailed by the Environmental Compliance and Enforcement Unit. The land invasions described related to the illegal occupation of land not zoned for residential purposes, whereby natural habitats were occupied and converted to residential properties. Both case studies were derived from respondents to the questionnaire.

### Ezemvelo KZN wildlife compliance-case study

To provide a case study of the experience of lockdown restrictions on a parastatal conservation organisation in South Africa, data on compliance in all protected areas managed by Ezemvelo KZN Wildlife (the parastatal conservation body in South Africa’s KwaZulu-Natal Province) were obtained for 2009 to 2020. Monthly data were obtained on the following offences: arson; damage to property; dog-related offences; firearms offences; human-wildlife conflict (livestock predation, conflict between humans and dangerous animals [i.e., those that cause damage to infrastructure and pose a danger to human life]); trespassing; illegal harvesting; poaching; permit offences; and other prohibited activities.

### Analyses of compliance data

A one-way Analysis of Variance (ANOVA) was performed in SPSS v27 (IBM Corp 2020) to investigate differences in the numbers of offences recorded per year, for all offences pooled, and for each respective offence in isolation. We used a Bonferroni adjustment and set the significance level at *P* < 0.01 to reduce type 1 errors associated with conducting 11 ANOVAs. A Tukey post-hoc test was performed in SPSS to investigate if the frequency of offences recorded in 2020 (during COVID-related lockdowns) differed from those in the previous ten years for all offences pooled and each offence in isolation. The case study was derived from Ezemvelo compliance data by one of the co-authors and respondents to the questionnaire.

### Monitoring of priority species by conservation NGOs- case study

To illustrate the impact of COVID-19 lockdown restrictions on the operations of non-governmental organisations (NGOs), we investigated the amount of time (in hours, representing conservation actions), and the distance travelled (in km, representing areas covered for conservation actions; Hilborn et al. 2006; Moore et al. 2017) performing conservation monitoring and intervention by the fieldworkers employed by Wildlife ACT, a conservation NGO focused on providing monitoring support for conservation partners (in particular, Ezemvelo KZN Wildlife) across South Africa’s KwaZulu-Natal Province. With six full-time monitoring teams in total, operating in six provincial and private nature reserves in KwaZulu-Natal, Wildlife ACT has a strong on-the-ground presence but relies heavily on a tourism-based model to fund its monitoring work. We compared the distance driven and time spent monitoring conservation priority species in 2019 and 2020. The case study was derived from respondents to the questionnaire.

## Results

### Questionnaire

**Fig. 1:**
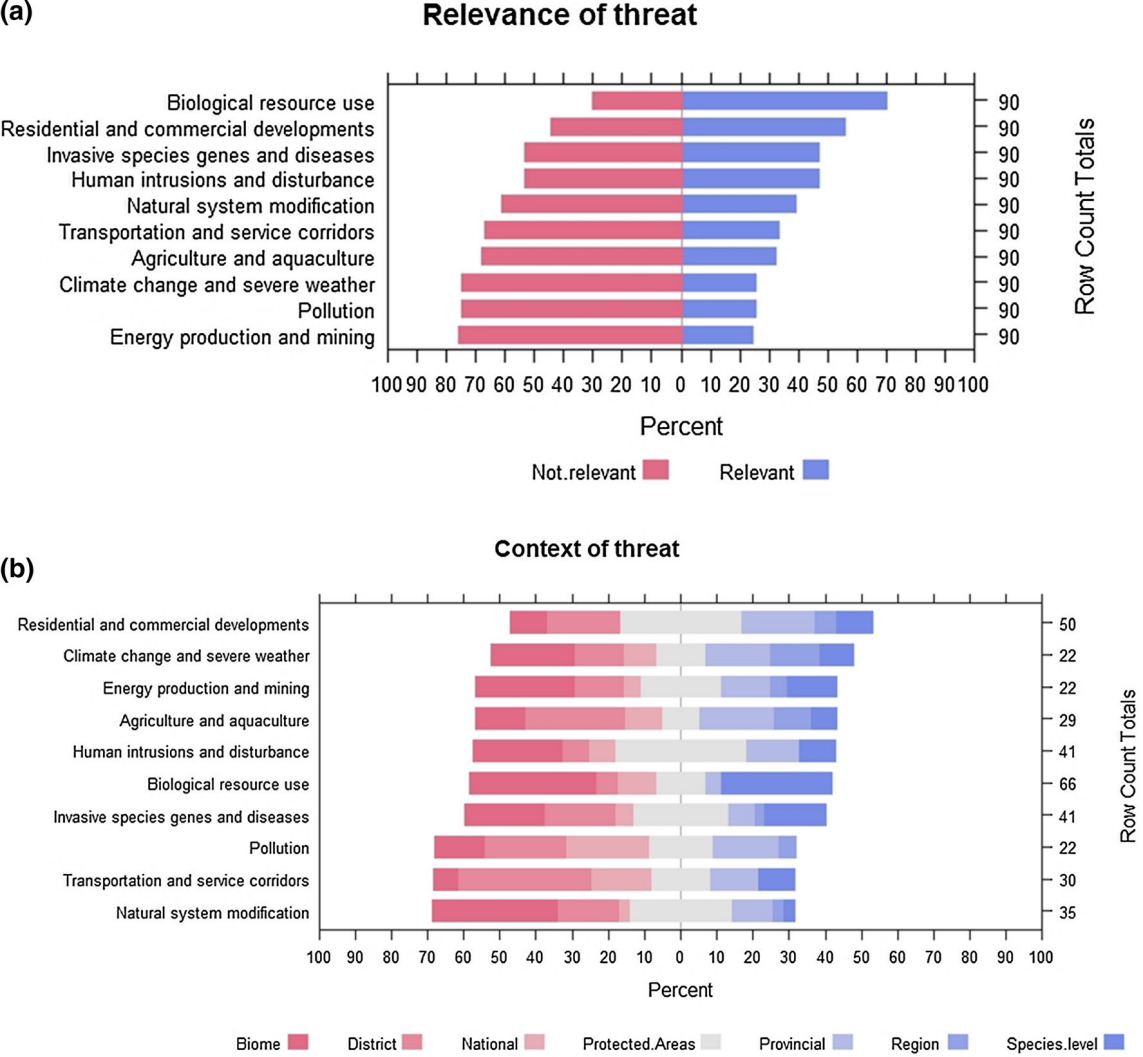
Frequency of reporting for how relevant each threat as derived from the IUCN’s Threats Classification Scheme was perceived to be during Covid-19 lockdown stages in South Africa from March 26th to December 28th 2020 (pane a) and context of all reported threats as derived from the IUCN’s Threats Classification Scheme during the lockdown levels in South Africa from March 26th to December 28th, 2020 (pane b). The colour classification in pane b is to distinguish between contexts

Our 90 survey respondents were from various conservation backgrounds, including 11 academic institutions, 25 NGOs, 13 parastatal departments and two consultancies. The questionnaire took a mean of 89 min. to complete. Respondents regarded the four highest-ranking threats to biodiversity during the COVID-19 lockdown period as biological resource use, residential and commercial developments, human intrusions and disturbance, and invasive species, genes and diseases. These threats were most frequently reported as being relevant, i.e., a threat applying to the context in question (Fig. 1a; Table 4). Biological resource use was reportedly the greatest threat at both the biome and species levels. For both levels, the threat increased in severity between lockdown levels 3–5 and lockdown levels 1–2. Threats from biological resource use were reportedly short-term, but there was little difference in the perception of changes to the severity of the threat when comparing biological resource use before and after respective lockdown levels (Table 4).

**Table 4:** Reporting rate frequency and percentage of respondents reporting relevance, context, direction, timescale and scope of the four highest-ranking threats to conservation during the COVID-19 lockdown in South Africa

		Biological resource use count (N)	Biological resource use %	Residential and commercial developments count (N)	Residential and commercial developments %	Human intrusion and disturbance count (N)	Human intrusion and disturbance %	Invasive species, genes and diseases count (N)	Invasive species, genes and diseases %
Relevant threat	Yes	63	70.0	50	55.6	42	46.7	42	46.7
	No	27	30.0	40	44.4	48	53.3	48	53.3
Context of threat	National	7	7.8	0	0.0	3	3.3	2	2.2
	Provincial	3	3.3	10	11.1	6	6.7	3	3.3
	Biome	23	25.6	5	5.6	10	11.1	9	10.0
	Region	0	0.0	3	3.3	0	0.0	1	1.1
	District	4	4.4	10	11.1	3	3.3	8	8.9
	Protected area	9	10.0	17	18.9	15	16.7	11	12.2
	Species level	20	22.2	5	5.6	4	4.4	7	7.8
	Not relevant	24	26.7	40	44.4	49	54.4	49	54.4
Direction of threat during lockdown levels 3–5	Threat increased	34	37.8	22	24.4	16	17.8	24	26.7
	Threat stable	5	5.6	4	4.4	0	0.0	3	3.3
	Threat reduced	16	17.8	14	15.6	23	25.6	5	5.6
	Threat suspected but unknown direction	6	6.7	3	3.3	3	3.3	3	3.3
	New threat	1	1.1	2	2.2	0	0.0	0	0.0
	Not relevant	28	31.1	45	50.0	48	53.3	55	61.1
Direction of threat during lockdown levels 1–2	Threat increased	36	40.0	19	21.1	21	23.3	19	21.1
	Threat stable	8	8.9	10	11.1	11	12.2	4	4.4
	Threat reduced	8	8.9	11	12.2	6	6.7	6	6.7
	Threat suspected but unknown direction	8	8.9	5	5.6	4	4.4	6	6.7
	New threat	2	2.2	0	0.0	0	0.0	0	0.0
	Not relevant	28	31.1	45	50.0	48	53.3	55	61.1
Timescale of threat during lockdown levels 3–5	Long-term (> 5 years)	12	13.3	17	18.9	3	3.3	8	8.9
	Medium-term (3–5 years)	19	21.1	9	10.0	9	10.0	11	12.2
	Short-term (duration of SoD)	22	24.4	17	18.9	27	30.0	8	8.9
	Unknown	8	8.9	2	2.2	3	3.3	7	7.8
	Not relevant	29	32.2	45	50.0	48	53.3	56	62.2
Timescale of threat during lockdown levels 1–2	Long-term (> 5 years)	12	13.3	15	16.7	4	4.4	8	8.9
	Medium-term (3–5 years)	18	20.0	9	10.0	15	16.7	8	8.9
	Short-term (duration of SoD)	19	21.1	14	15.6	15	16.7	10	11.1
	Unknown	12	13.3	5	5.6	3	3.3	8	8.9
	Not relevant	29	32.2	47	52.2	53	58.9	56	62.2
Scope of threat before lockdown	Affects the whole (> 90%)	6	6.7	4	4.4	2	2.2	6	6.7
	Affects the majority (50–90%)	20	22.2	12	13.3	15	16.7	10	11.1
	Affects the minority (< 50%)	32	35.6	21	23.3	21	23.3	14	15.6
	Unknown	4	4.4	8	8.9	4	4.4	5	5.6
	Not relevant	28	31.1	45	50.0	48	53.3	55	61.1
Scope of threat during lockdown levels 3–5	Affects the whole (> 90%)	8	8.9	10	11.1	4	4.4	7	7.8
	Affects the majority (50–90%)	23	25.6	14	15.6	15	16.7	13	14.4
	Affects the minority (< 50%)	23	25.6	16	17.8	20	22.2	8	8.9
	Unknown	8	8.9	5	5.6	3	3.3	7	7.8
	Not relevant	28	31.1	45	50.0	48	53.3	55	61.1
Scope of threat during lockdown levels 1–2	Affects the whole (> 90%)	8	8.9	8	8.9	3	3.3	7	7.8
	Affects the majority (50–90%)	24	26.7	18	20.0	16	17.8	11	12.2
	Affects the minority (< 50%)	23	25.6	11	12.2	16	17.8	9	10.0
	Unknown	7	7.8	8	8.9	7	7.8	8	8.9
	Not relevant	28	31.1	45	50.0	48	53.3	55	61.1

Threats to biodiversity from residential and commercial developments were frequently reported in the context of protected areas, and at the provincial and district levels. This threat reportedly increased at the strictest COVID-19 lockdown levels, despite the severe restrictions on human movement, and it was regarded as potentially having long-term impacts with a greater impact after than before lockdown restrictions were implemented (Table 4).

The threat of ‘human intrusion and disturbance’ to biodiversity was most pronounced in the context of protected areas and biomes. This threat was perceived to diminish at the strictest COVID-19 lockdown levels and increase again after the easing of lockdown restrictions. Respondents perceived the threat to be short-term. There was little change in the perceived scope of human intrusion threats before and during COVID-19 lockdown restrictions (Table 4). An increase in the threat of ‘invasive species, genes and diseases’ was reported after lockdown restrictions eased, particularly in the context of protected areas. There was no clear trend in the duration or scope of threats in relation to the various levels of COVID-19 lockdown restrictions (Table 4).

The threat from residential and commercial developments and human intrusions was mostly relevant to protected areas, and at the biome level (Fig. 1b). A summary of the direction, scope and timescale of the less frequently reported threats may be found in the Supplementary Information S2.

### Case study I: Illegal hunting with dogs in South Africa’s Eastern Cape Province

Data from the Eastern Cape Province’s Environmental Compliance and Enforcement Unit revealed that from January 2019 to February 2020 (pre-lockdown), four cases of illegal hunting with dogs were recorded in the Chris Hani Municipality, Eastern Cape. From the onset of the COVID-19 lockdown until December 2020, 45 incidents of illegal hunting with dogs were reported, representing a 1025% increase in the frequency of hunting events per month from pre-lockdown to during lockdown. In the Joe Gqabi District, four cases of illegal hunting with dogs were recorded in 2019, increasing to 10 cases in 2020. In the Amathole District, where more detailed categories of offences committed were available, the number of complaints of illegal hunting with dogs that were attended to increased by 130% from 2019 to 2020, with arrest and conviction rates varying between the two years but with no clear trends (Table 5).

**Table 5:** Reporting frequencies of illegal poaching with dogs in the Amathole District of Eastern Cape Province, South Africa, in the year preceding COVID-19 lockdown and during lockdown

Key result area	2019	2020
Number of complaints attended	20	46
Number of cases opened	17	22
Number of people arrested	57	40
Number of minors involved	31	22
Number of dogs impounded	40	21
Number of animal carcass seized	9	10
Number of vehicles involved	5	7
Number of vehicles confiscated	3	7
Number of convictions	3	1
Written and Verbal warnings issued	16	22

### Case study II: Illegal land invasions in South Africa’s Eastern Cape Province

The Grey Dell Forests are declared Controlled Forests and Natural Forests by the Minister of Forestry, Fisheries and the Environment, adjacent to the Umtiza Nature Reserve and the King Phalo Airport in East London and are a portion of state farmland. Before 2019, these indigenous forests on the farmland were not invaded, and the intergovernmental task team established by the Department of Public Works in 2015 recommended that they be declared natural forests and amalgamated with the Umtiza Nature Reserve to be managed by the Eastern Cape Parks and Tourism Agency. In 2019, the clearing of indigenous forests by settlers began on the property that the intergovernmental task team had surveyed for declaration, and by the end of the year, seven shacks had been erected on this portion of land. The High Court Order to demolish unlawful structures was implemented in July 2020, during the middle of the COVID-19 pandemic and lockdown restrictions. The court granted an interdict to the settlers to prevent the state from continuing with the demolition order based on allegations that the settlers were occupying these houses and had no other residences. The court ruled that the state needed to provide alternative housing. This prevented the state from demolishing the few settlements that had been built in the indigenous forest by low- and high-income settlers. The court order that was granted to the settlers opened the way for increasing settlements within the indigenous forest. The state appealed the court interdict; however, various stages of COVID-19 lockdown and accompanying legislation prevented the eviction of people from any land during the pandemic (DPW 2021). When these laws became common knowledge, rampant land invasions followed on the property. In line with the intergovernmental task team’s recommendations, the Grey Dell indigenous forest was gazetted as a declared natural forest by the Minister of Forestry, Fisheries and the Environment in December 2020, but this did not prevent forest invasions. A judge subsequently ruled that the state broke the law relating to Level 3 COVID-19 legislation, which prohibited evictions by demolishing houses at Airport Park in Grey Dell. The judge ordered the state to rebuild 77 houses that had been demolished during the July operation because of the judgement (which referred to COVID-19 legislation) that opened the doors to further invasions and prevented law enforcement officials from implementing demolitions and evictions. By December 2020, more than 200 houses had been erected in the forest on the farm, and the settlers had unlawfully cleared approximately 50% of the 250 ha forested area.

### Case study III: Environmental offences recorded in South Africa’s KwaZulu-Natal Province

**Fig. 2:**
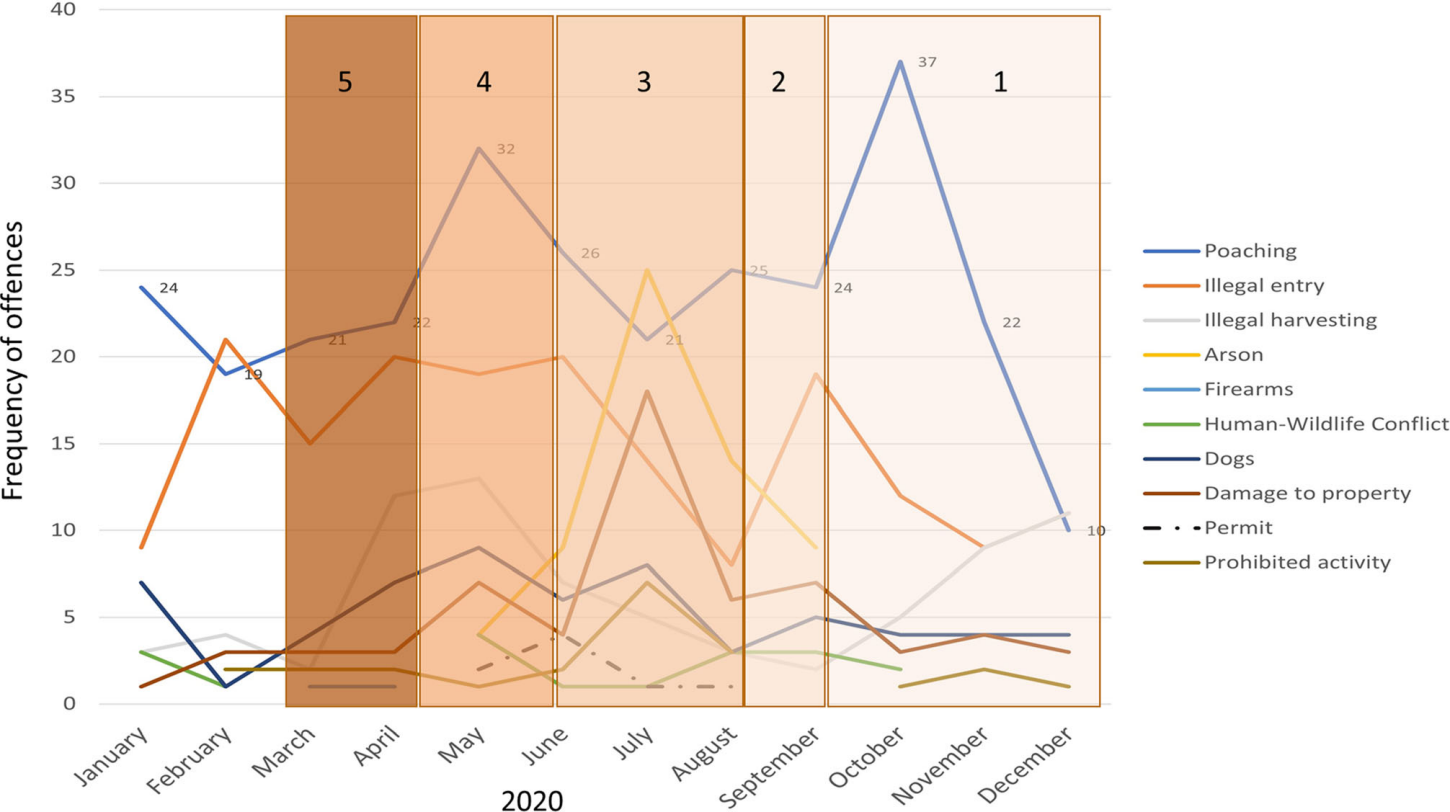
Trends in offences recorded during the five stages of lockdown by Ezemvelo KwaZulu-Natal Wildlife Compliance in KwaZulu-Natal Province, South Africa. Stage numbers are detailed horizontally

The monthly registered relevant offences, as collated by Ezemvelo KZN Wildlife, are shown in Fig. 2. The timeframe spanned the pre-pandemic months (January to March 2020), followed by the COVID-19 lockdown restriction levels 5 through to 1. By these parameters, the number of infringements was expected to decrease during the strictest lockdown periods (when people’s movements were most heavily curtailed) compared with pre-lockdown, increasing as lockdown restrictions eased. Illegal entry into Protected Areas appeared to show a downward trend during the onset of lockdown restrictions, but offences soon increased after the strictest levels eased. Other recorded offences showed no clear pattern linked to lockdown levels and associated restrictions.

**Fig. 3:**
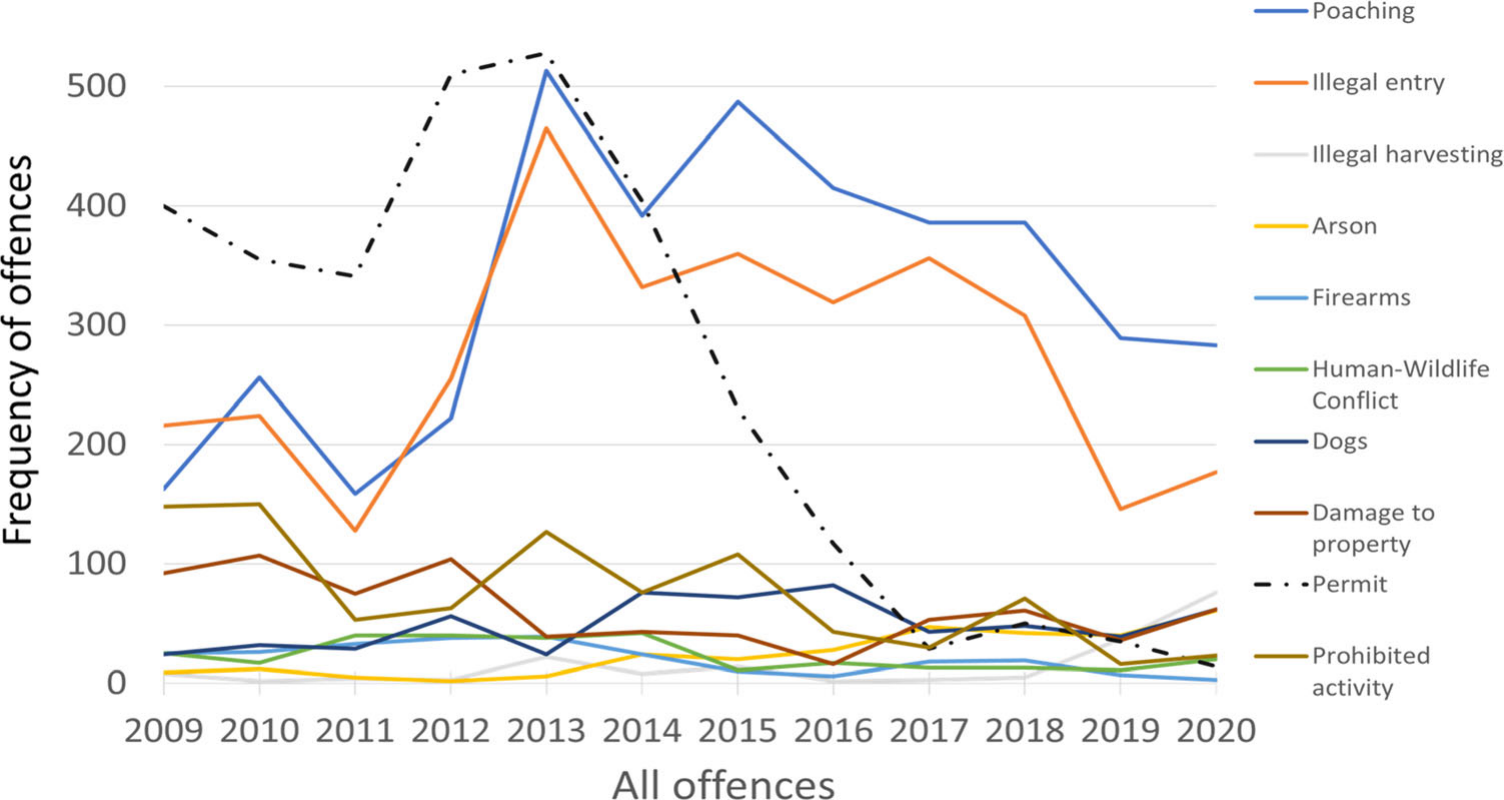
All offences recorded by Ezemvelo KwaZulu-Natal Wildlife Compliance between 2009 and 2020 in KwaZulu-Natal Province, South Africa

Results from the comparison of the number of individual offences over the 11-year dataset indicated significant differences between 2020 and previous years individually for illegal hunting (one-way ANOVA, *F*_143_ = 6.22; *P* < 0.001; Tukey post-hoc, 2013 = *P* < 0.05); illegal entry (*F*_143_ = 7.01; *P* < 0.001; Tukey post-hoc: 2013 and 2017 = *P* < 0.05); harvesting (*F*_143_ = 13.27; *P* < 0.001; Turkey post-hoc: all years = *P* < 0.05); firearms (*F*_143_ = 5.16; *P* < 0.001; Tukey post-hoc: 2011, 2012 and 2013 = *P* < 0.05); human-wildlife conflict (*F*_143_ = 4.18; *P* < 0.001; Tukey post-hoc: no significant difference between 2020 and other years); hunting with dogs (*F*_143_ = 4.64; *P* < 0. 001; Tukey post-hoc: no significant difference between 2020 and other years); damage to property/vandalism (*F*_143_ = 3.17; *P* < 0. 001; Tukey post-hoc: no significant difference between 2020 and other years); permit infringements (*F*_143_ = 20.78; *P* < 0.001; Tukey post-hoc: 2011–2015, *P* < 0.05); prohibited activities (*F*_143_ = 6.90; *P* < 0.001; Tukey post-hoc: 2009 2010, 2013 and 2015, *P* < 0.05), and arson (*F*_143_ = 1.86; *P* < 0.05; Tukey post-hoc: no significant difference between 2020 and other years) (Fig. 3).

**Fig. 4:**
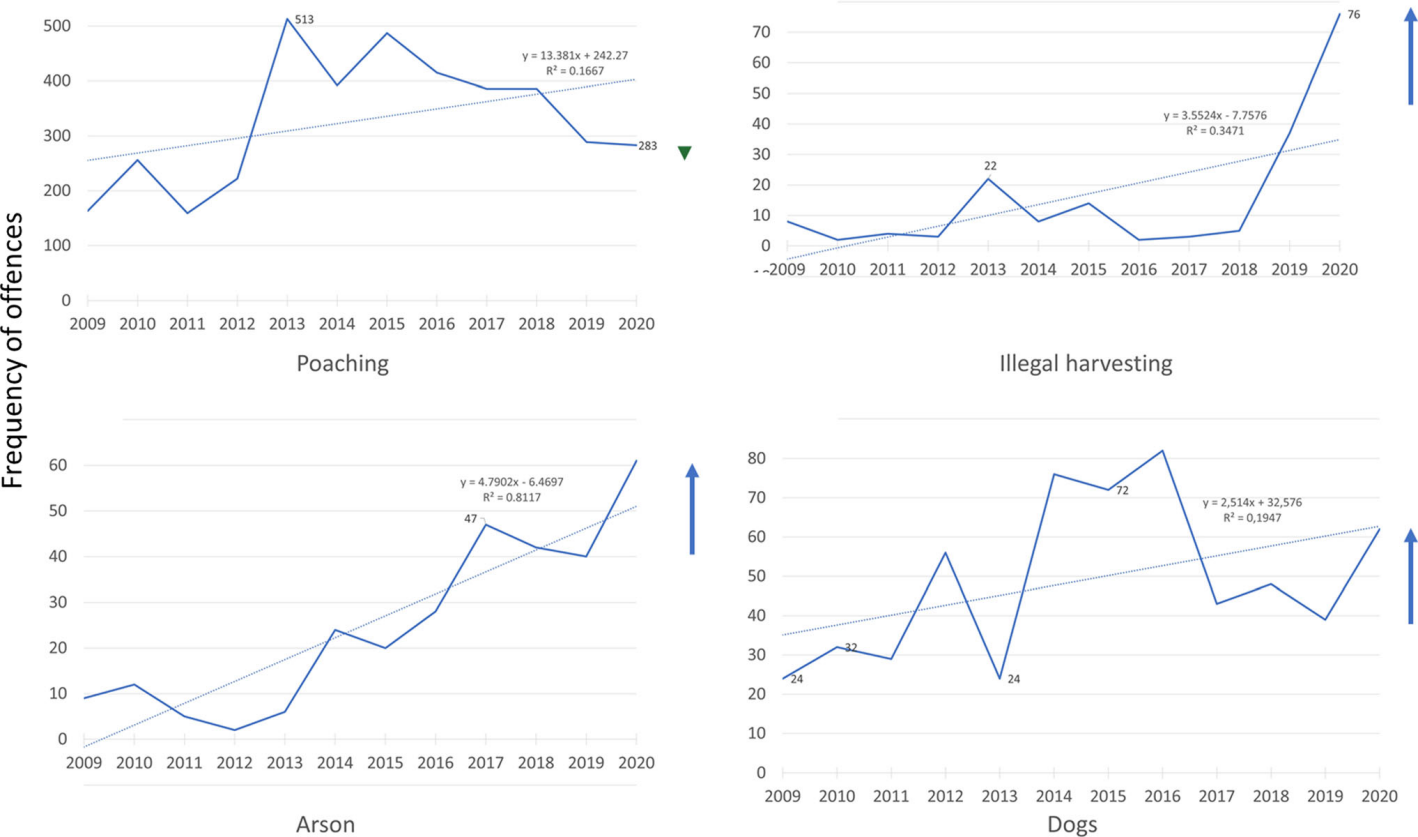
Trends of increasing offences from 2009 to 2020 as recorded by Ezemvelo KwaZulu-Natal Wildlife in KwaZulu-Natal Province, South Africa

Between 2009 and 2020, illegal hunting was the most recorded of the various incidents (30%), while illegal plant harvesting comprised only 2% of incidents. For some offences, the overall trends increased, including poaching (*R*^2^ = 0.17), illegal harvesting of natural resources (*R*^2^ = 0.35), arson fires (*R*^2^ = 0.81), and the presence of dogs in Protected Areas (*R*^2^ = 0.20; Fig. 4).

### Case study IV: Priority species monitoring by a conservation NGO

**Fig. 5:**
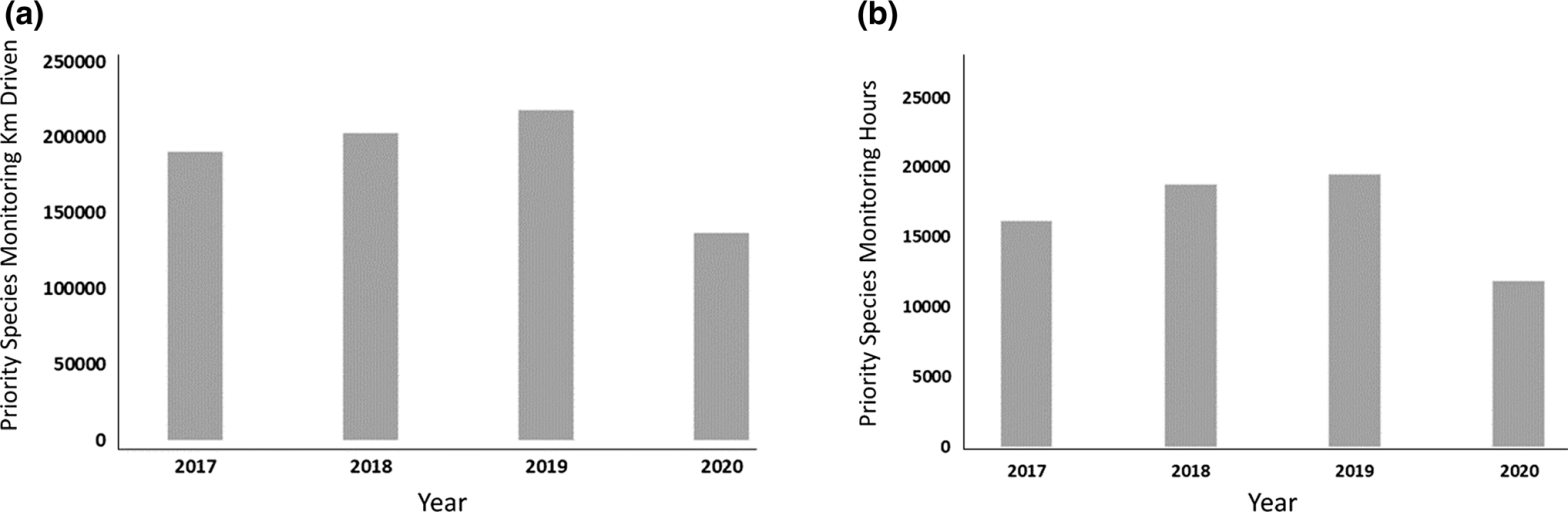
Conservation priority species monitoring in protected areas of KwaZulu-Natal Province, South Africa by non-governmental organisation Wildlife Act. Pane **a** shows kilometres driven between 2017 and 2020 to monitor conservation priority species, and Pane **b** shows hours spent monitoring conservation priority species in KwaZulu-Natal

With the onset of COVID-19 in 2020 and the restrictions on people’s movements (and consequently voluntourism), Wildlife ACT’s funds for priority species monitoring were severely reduced. In response, the monitoring teams were required to reduce fuel expenditure and their time in the field. As a result, in 2020, there was a 37% reduction (of 81,798 km) in the distance-driven performing conservation monitoring and interventions and a 39% reduction (7565 h) in time spent in the field performing conservation monitoring and interventions inside protected areas, when compared with 2019 (Fig. 5). This contrasts greatly with the successive increase in effort (as measured in h and km) over the previous three years.

## Discussion

In the face of persistent poverty, high unemployment levels (35.3% in 2021; Statistics SA), extreme social inequality, and environmental pressures resulting from increasing human populations, our results showed that the COVID-19 pandemic and the lockdown restrictions implemented by the South African government further enabled and exacerbated the country’s pre-existing reliance on biological resources, as predicted. The most highly-reported threats to biodiversity, with the highest-ranking derived threat categories, were biological resource use, threats from residential and commercial developments, invasive species, and human intrusions (Fig. 1a; Table 4). These threats were particularly highly reported as increasing during lockdown restrictions at the species-level, protected area, and biome levels (Fig. 1b). This suggests that illegal intrusions into protected areas occurred for the purpose of resource extraction, and invasions into protected areas and biomes happened during lockdown restrictions when law enforcement agencies were not operating at their maximum capacities. Overall trends suggested these threats had the potential to be long-term, but many were in the context of lockdown or short-to-medium term, with a medium risk scope (Table 4; Supplementary Information S2). These results supported our prediction that a closed economy would increase resource extraction as job losses meant that people struggled to meet their basic daily needs.

The trends reported by our survey respondents were supported by several case studies using the data from parastatal conservation organisations regarding compliance with environmental laws (Figs. 3, 4, and 5; Table 5). In the Eastern Cape Province, a dramatic increase in illegal hunting with dogs was reported across several regions during the lockdown (De Villiers, 2020; Table 5). We described a case of illegal land invasions of private property outside of East London, where biological resource extraction and natural forest clearing took place. This highlighted the impact of COVID-19 lockdown restrictions on legal procedures concerning land rights and protected area conservation and the subsequent exploitation of the legal situation during lockdown phases. These results and case studies supported our predictions that a reduction in funds and law enforcement would decrease adequate conservation monitoring and enforcement.

Our survey respondents provided evidence further highlighting the increase in harvesting of specific species directly because of the nature of the COVID-19 pandemic and its effects on human health and societal responses in attempting to mitigate adverse health effects. For example, during the pandemic, *Artemesia afra*, a South African endemic plant, was touted by traditional healers as an effective traditional medicine for treating breathing difficulties and, therefore, a potential cure for COVID-19. Increases in the harvesting and sale of this plant were reported across rural landscapes of the Eastern Cape Province and in traditional medicine markets (D. Rickets, pers. obs.). The species is not protected in the Eastern Cape; however, the explosive demand and collection of the plant prompted the Eastern Cape Department of Economic Development, Environmental Affairs and Tourism to consider implementing measures to prevent its unsustainable use, and the continued pandemic exacerbated the issue, until its decline in popularity as a cure. Other taxa reported in the questionnaire to be particularly at threat during the pandemic were ungulates that are vulnerable to snaring, large terrestrial birds and vultures (Accipitridae, which were undergoing catastrophic declines in Africa even before the COVID-19 pandemic, Ogada et al. 2016; Fig. 1b).

In KwaZulu-Natal Province, despite the severe movement restrictions imposed during the level 5 strictest COVID-19 lockdown level, the only offences recorded by Ezemvelo KZN Wildlife for which cases decreased during this period were illegal entries into protected areas and illicit activities (driving on beaches, disembarking from vehicles in game parks etc.), which may have been because field rangers (law enforcement officers) were still active in protected areas as an essential service. Conversely, the reduced presence of park personnel in the field (there were operational restrictions during lockdown despite the protection of nature being regarded as an essential service and granted special permissions for personnel to operate during lockdown) may have detected fewer cases of illegal entry. Similarly, law enforcement also relies on tourists and tourism operators as “whistleblowers” for many infringements that occur within the Protected Area Network. A conservation NGO (Wildlife ACT), which is reliant on tourism for its operational budget, reduced its distances travelled and time spent monitoring species of conservation priority (Fig. 5), which supported our first prediction that reduced tourism and tourism revenue (including that from hunting) would be detrimental to wildlife conservation.

Overall, trends over the past decade suggested that wildlife offences were declining (with the exception of permit offences, where the change was a result of changes to the specific offences that were being recorded by Ezemvelo KZN Wildlife staff as mandated [Y Ehlers Smith, pers. obs.]). However, an increase in most offences was reported for 2020 (Fig. 3). Furthermore, there were significant differences in the frequencies of some offences (e.g., illegal harvesting, arson, and illegal hunting with dogs) between 2020 and previous years (Fig. 4). Illegal hunting, arson (corresponding with the austral dry/winter season) and illegal entry offences increased (Fig. 2) as citizens were allowed greater freedom of movement after COVID-19 lockdown restrictions of levels 3 to 5 were eased during levels 1 and 2. This supported predictions 2 and 3 that reduced funding for conservation safeguards and a closed economy would increase wildlife consumption. However, it is worth noting that the trend of arson was increasing before the lockdown as a result of poor grassland management and increased pressure for utilisation (Ehlers Smith et al. 2021). Testimonies from experts in the KwaZulu-Natal conservation sector were reported in the questionnaire responses. Respondents mentioned hindrances to conservation monitoring and enforcement, decreased funding and fewer personnel to implement conservation activities effectively and enforce environmental compliance. Anecdotal evidence suggested an increase in the setting of snares in the Hawaan Private Nature Reserve and Amanzimtoti residential areas during the lockdown, after previous successes of snare eradication programmes during preceding years. This supported our second prediction that conservation research and safeguards, including the maintenance and allocation of funds to conduct practical conservation interventions, would be negatively impacted by reduced government funding during the pandemic, and by restrictions on movements and access to natural areas. This anecdotal evidence was presented with limitations, as monitoring data were not available from the majority of protected areas nationwide. However, according to our questionnaire respondents across the country, a hindrance to movements and a lack of funding were reported across all sectors involved in biodiversity conservation, a phenomenon echoed by a recent pre-pandemic horizon scan of conservation issues in South Africa, which warned of a diversion of conservation funding and a bypassing of environmental laws during crises and states of emergency (Seymour et al. 2019). These results supported predictions one to three.

In 2011 the South African hunting industry generated ~ R7.7 billion (~ US$500 million, at the present exchange rate of 1 USD = 15.39 ZAR, www.xe.com), which equated to 25% of South Africa’s national Gross Domestic Product for that year, which ~ R2.1 billion p.a. was generated from international hunters visiting South Africa (pers. comm to DR via E. Rudman, 2020). According to the Professional Hunters Association of South Africa, as much as 90% of the income brought in by international hunters was lost in 2020 because of the stricter lockdown conditions (pers. comm. to DR via E. Rudman, 2020). The hunting industry was further affected by the prohibition of interprovincial travel and limits on the utilisation of overnight accommodation. Local hunters make up the largest portion of hunting clients and contribute significant revenue to the hunting industry and South Africa’s economy (pers. comm. to DR via E. Rudman 2020).

In the face of the highest unemployment levels ever recorded in South Africa (35.3% of the population; Statistics SA 2022), such large-scale economic and job losses place further pressure on a struggling economic sector, which in turn further threatens biodiversity resources and the informal protection of wild spaces (Taylor et al. 2019). This supported our first prediction that a reduction in income from tourism would negatively affect conservation in South Africa. South Africa’s Department of Forestry, Fisheries and Environment highlighted the substantial shortfall in income during the lockdown for their four main entities: South African National Parks, the South African National Biodiversity Institute, iSimangaliso Wetland Park Authority, and the South African Weather Service. These funding shortfalls impacted the abilities of these four entities to remain self-sustaining (DFFE 2021). In response, the South African government has shifted R1.1 billion Rand (~ US$75 million) to these entities to reduce their funding shortfalls and ensure their operations are sustainable and secured (DFFE 2021). This is an encouraging example of government funding being allocated to mitigate economic shortfalls of conservation/environmental entities after the initial crisis, but it also highlighted the vulnerability of the environmental/biodiversity sector during national emergencies.

An additional complication of the COVID-19 pandemic is the loss of human capacity. For example, over a third of the Eastern Cape’s Compliance and Enforcement Unit’s Environmental Management Inspectors had contracted COVID-19 by the end of 2020, and two Compliance and Enforcement officers and an Environmental Management Inspector passed away because of complications from the disease, while as of December 2020, several officials are still fighting the long-term effects of COVID-19 (pers. comm.). These were all frontline workers who participated in compliance monitoring inspections, enforcement investigations, and operations. Whereas most line function staff have stayed at home, Environmental Management Inspectorates have continued working in the field throughout the COVID-19 lockdown periods and were exposed to higher risks of contracting the disease. In addition to personal tragedy, these represent a critical loss of skills and knowledge in the sector.

## Conclusions

Globally, the pandemic has been framed as an opportunity for systemic change in the conservation sector, including recognition of how quickly established institutions and practices may adapt their work in response to a crisis to elevate the importance of biodiversity conservation in political agendas (Thurstan et al. 2021). Cross-sectoral engagement is possible if there is political and economic will (Lindsey et al. 2020; Roe et al. 2020). The South African experience of the fate of conservation during a global crisis, as highlighted by our results, has been echoed across most countries, suggesting that global attitudes to conservation may change if local experiences can be represented, prioritised, and incorporated into national and global agendas, and if lasting and meaningful cross-sectoral relationships can be established (Miller-Rushing et al. 2021). However, the immediate global (and South African) response was to divert funds from the conservation sector to immediate disaster mitigation and management in relation to human needs. This suggests that governments’ recognition of the critical nature of biodiversity conservation in such times of crisis is a long way from realisation.

The success of future conservation research, monitoring, and implementation hinges on our desire to learn from the lessons and threats that the COVID-19 pandemic has highlighted (Schwartz et al. 2020; Thurstan et al. 2021; Gibbons et al. 2022). Gibbons et al. (2022) identified reduced governmental and philanthropic funding as among the top ten threats to biodiversity conservation worldwide following the COVID-19 pandemic. Therefore, in South Africa, funding for national and provincial policy change and implementation, conservation research, monitoring, and sufficient funding allocation to implement actions must be recognised as critical components of our country’s ongoing climate change and biodiversity decline mitigation. Safeguards to prevent environmental degradation in the face of global emergencies must be implemented and ‘ring-fenced’ to ensure that biodiversity conservation does not again become a casualty of future global crises.

## Supplementary Information

Below is the link to the electronic supplementary material.

Supplementary file1 (PDF 7551 kb)

## Data Availability

The data belong to the University of KwaZulu-Natal and the various provincial conservation bodies and non-government organisations. These data are available from the corresponding author upon reasonable request.

## Footnotes

1 https://www.iucnredlist.org/resources/threat-classification-scheme.

2 www.forms.office.com.
