# Supplementary material for: The impacts of a global pandemic on the efficacy and stability of contemporary wildlife conservation: South Africa as a case study
Source: Ambio. 2022 Dec 30;52(3):598–615. doi: 10.1007/s13280-022-01814-z (PMC9802021; doi:10.1007/s13280-022-01814-z)
Supplement: Supplementary file 1 — Supplementary file1 (PDF 7551 kb) [file 13280_2022_1814_MOESM1_ESM.pdf]

Ambio
Electronic Supplementary Material
This supplementary material has not been peer-reviewed.

Title: **The impacts of a global pandemic on the efficacy and stability of contemporary wildlife conservation: South Africa as a case study**

Authors: David A. Ehlers Smith, Yvette C. Ehlers Smith, Harriet T. Davies-Mostert, Lindy J. Thompson, Daniel M. Parker, Deon De Villiers, Dean Ricketts, Brent Coverdale, Peter J. Roberts, Christopher Kelly, Duncan N. Macfadyen, Nomthandazo S. Manqele, R. John Power, & Colleen T. Downs

Supplementary information Table S1. Questionnaire used in the present study.

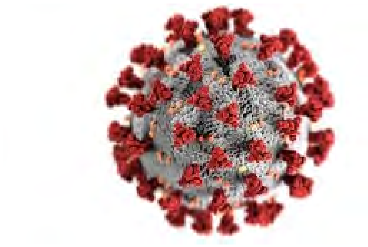

Impacts of Covid-19 Lockdown Restrictions on Biodiversity and its Conservation in SA

Dear recipient,

We wish to solicit your expert advice regarding the impacts of the Covid-19 pandemic and the associated lockdown restrictions, on biodiversity and its conservation in South Africa. You have been highlighted as a possible contributor of information toward a paper that aims to assess potential changes to biodiversity and its conservation as affected by altered threats during lockdown. Your contribution is not anonymous and will be acknowledged according to prior agreement with the steering committee.

We are have selected the IUCN's Threats Classification Scheme (https://www.iucnredlist.org/resources/threat-classification-scheme (https://www.iucnredlist.org/resources/threat-classification-scheme)) as a framework to investigate how the pandemic and movement restrictions affected biodiversity and its conservation in South Africa. We provide a framework to guide your expert advice on the following pages; the threat categories are as follows:

- Residential and commercial developments
- Agriculture and aquaculture
- Energy production and mining
- Transportation and service corridors
- Biological resource use
- Human intrusion and disturbance
- Natural system modification
- Invasive species, genes and diseases
- Pollution
- Climate change and severe weather

You have the option to skip a threat/section if not applicable.

Consider your answers within the context of your area of expertise. You have the opportunity to name your area of expertise; if you have multiple fields of expertise that have been affected in different ways, you are free to either choose one, or repeat the questionnaire.

Consider your answers within the context of "lockdown" restrictions on movement, measures to prevent spread of the virus (mandatory mask wearing/sanitising/restrictions on gatherings), classification of essential services and the introduction of permitted activities between Levels 5 to 3 and eased restrictions between Levels 2 to 1:

Lockdown Level 5 = March 26 - May 01 2020 ("Hard lockdown" - only essential services operating: health workers, pharmacy and laboratory personnel, emergency personnel; security services (police officers, military personnel, and private security; people regarded as necessary to the basic functioning of the economy (supermarkets, transportation and logistical services, petrol stations, banks, essential financial and payment services); and those working in industries that can not be economically shut down (such as mines and steel mills))

Lockdown Level 4 = May 02 - June 01 2020 (Local, provincial and international travel banned, services opened for food, cleaning, protective, babycare, stationery; winter clothing, bedding, heating; medical supplies; fuel, coal, wood, gas; hardware supplies for emergency home repairs and essential services by qualified tradespersons; components for vehicles for essential workers; chemicals, packaging, and supply of level 4 products; curfew 20:00-05:00)

Lockdown Level 3 = June 02 - August 17 2020 (Local, provincial and international travel banned, places closed to public, all businesses may operate except liquor and tobacco retailers; short-term home rental for leisure purposes; passenger ships for leisure purposes; and entertainment activities; reserves open for self-drive reserves but overnight guests prohibited; curfew 21:00-04:00)

Lockdown Level 2 = August 18 - September 21 2020 (Inter-provincial travel ban lifted, international travel limitations, gathering restrictions eased and gyms open, overnight accommodation permitted, reserves open for overnight guests; curfew 22:00-04:00)

Lockdown Level 1 = September 22 - December 28 2020 (curfew 0:00-04:00)

Adjusted Lockdown Level 3 = December 29 2020 - ongoing (curfew 21:00-05:00, restrictions on certain economic sectors)

We thank you for offering your expertise and experiences.
Kind regards,
Colleen Downs, Harriet Davies-Mostert, Daniel Parker, Lindy Thompson, Yvette Ehlers Smith & David Ehlers Smith

For queries or feedback, please contact Prof Colleen Downs: downs@ukzn.ac.za (mailto:downs@ukzn.ac.za)

* Required

Personal information

By filling out your personal information in this section you confirm that you understand the contents of this document and the nature of the research project, and that you consent to participating in the research project. You are at liberty to withdraw from the project at any time, should you desire. This is not an anonymous procedure because we may need to follow up for data verification purposes or further quantitative data may be requested. A copy of the published article will be shared with all contributors.

1. Name *

2. Email address *

3. Affiliation(s) *

4. Area of expertise *

Impacts of RESIDENTIAL AND COMMERCIAL DEVELOPMENTS

These are threats tied to a defined and relatively compact area, which have a long narrow footprint, including but not limited to:
Housing and urban areas;
Commercial and industrial areas;
Tourism and recreation areas;
Legal and illegal developments (e.g. land invasions)

5. Is this threat relevant to your area of expertise? If not, please tick "Not relevant" to go to the next section *

- ◯ Relevant
- ◯ Not relevant

6. Please provide the name of the species/community/habitat/ecosystem/locality (e.g. reserve)/bioregion (e.g. Lowveld) you are referring to for the above question *

7. Did lockdown restrictions have any influence on the impacts of RESIDENTIAL AND COMMERCIAL DEVELOPMENTS on the species/community/habitat/ecosystem/locality (e.g. reserve)/bioregion (e.g. Lowveld) for which you have expertise? *

- ◯ Yes
- ◯ Suspected/likely
- ◯ No
- ◯ Unsure

8. How did the lockdown restrictions influence the above threat to biodiversity the species/community/habitat/ecosystem/locality (e.g. reserve)/bioregion (e.g. Lowveld) for which you have expertise? *

	Threat reduced	Threat increased	Threat stable	Impact on threat suspected but direction unknown	New threat
Lockdown levels 3 to 5	○	○	○	○	○
Lockdown levels 1 to 2	○	○	○	○	○

9. How long do you expect the effects of the lockdown restrictions on the above threat to last? *

	Short-term (for the duration of the State of Disaster)	Medium-term (3-5 years)	Long-term (>5 years)	Unknown	N/A
Lockdown levels 3 to 5	○	○	○	○	○
Lockdown levels 1 to 2	○	○	○	○	○

10. What was the scope of the above threat for the species/community/habitat/ecosystem /locality (e.g. reserve)/bioregion (e.g. Lowveld) for which you have expertise IMMEDIATELY BEFORE LOCKDOWN? *

	Affects the minority of the population/ community/ habitat/ ecosystem, etc. (<50%)	Affects the majority of the population/ community/ habitat/ ecosystem, etc. (50-90%)	Affects the whole population/ community/ habitat/ ecosystem, etc. (>90%)	Unknown
Immediately before lockdown	○	○	○	○

11. What is the scope of the above threat as a result of lockdown restrictions for the species/community/habitat/ecosystem/locality (e.g. reserve)/bioregion (e.g. Lowveld) for which you have expertise? *

	Affects the minority of the population/ community/ habitat/ ecosystem, etc. (<50%)	Affects the majority of the population/ community/ habitat/ ecosystem, etc. (50-90%)	Affects the whole population/ community/ habitat/ ecosystem, etc. (>90%)	Unknown
Lockdown levels 3 to 5	○	○	○	○
Lockdown levels 1 to 2	○	○	○	○

12. Please provide details of the effects you have reported above, including any data or evidence you have access to (indicate "N/A" if none) *

The impacts of AGRICULTURE AND AQUACULTURE

These threats are from farming, ranching and plantations as a result of agricultural expansion and intensification, including silviculture, mariculture and aquaculture (includes the impacts of any fencing around farmed areas), including but not limited to:
Annual and perennial non-timber crops ;
Wood and pulp plantations;
Livestock farming and ranching;
Marine and freshwater aquaculture

13. Is this threat relevant to your area of expertise? If not, please tick "Not relevant" to go to the next section *

◯ Relevant

◯ Not relevant

14. Please provide the name of the species/community/habitat/ecosystem/locality (e.g. reserve)/bioregion (e.g. Lowveld) you are referring to for the above question *

15. Did lockdown restrictions have any influence on the impacts of AGRICULTURE AND AQUACULTURE on the species/community/habitat/ecosystem/locality (e.g. reserve)/bioregion (e.g. Lowveld) for which you have expertise? *

◯ Yes

◯ Suspected/likely

◯ No

◯ Unsure

16. How did the lockdown restrictions influence the above threat to the species/community /habitat/ecosystem/locality (e.g. reserve)/bioregion (e.g. Lowveld) for which you have expertise? *

	Threat reduced	Threat increased	Threat stable	Impact on threat suspected but direction unknown	New threat
Lockdown levels 3 to 5	○	○	○	○	○
Lockdown levels 1 to 2	○	○	○	○	○

17. How long do you expect the effects of the lockdown restrictions on the above threat to last? *

	Short-term (for the duration of the State of Disaster)	Medium-term (3-5 years)	Long-term (>5 years)	Unknown	N/A
Lockdown levels 3 to 5	○	○	○	○	○
Lockdown levels 1 to 2	○	○	○	○	○

18. What was the scope of the above threat for the species/community/habitat/ecosystem /locality (e.g. reserve)/bioregion (e.g. Lowveld) for which you have expertise IMMEDIATELY BEFORE LOCKDOWN? *

	Affects the minority of the population/ community/ habitat/ ecosystem, etc. (<50%)	Affects the majority of the population/ community/ habitat/ ecosystem, etc. (50-90%)	Affects the whole population/ community/ habitat/ ecosystem, etc. (>90%)	Unknown
Immediately before lockdown	○	○	○	○

19. What is the scope of the above threat as a result of lockdown restrictions for the species/community/habitat/ecosystem/locality (e.g. reserve)/bioregion (e.g. Lowveld) for which you have expertise? *

	Affects the minority of the population/ community/ habitat/ ecosystem, etc. (<50%)	Affects the majority of the population/ community/ habitat/ ecosystem, etc. (50-90%)	Affects the whole population/ community/ habitat/ ecosystem, etc. (>90%)	Unknown
Lockdown levels 3 to 5	○	○	○	○
Lockdown levels 1 to 2	○	○	○	○

20. Please provide details of the effects you have reported above, including any data or evidence you have access to (indicate "N/A" if none) *

The impacts of ENERGY PRODUCTION AND MINING

These threats are from production of non-biological resources, including but not limited to:
Oil and gas drilling;
Mining and quarrying;
Renewable energy;
Legal and illegal prospecting and exploitation

21. Is this threat relevant to your area of expertise? If not, please tick "Not relevant" to go to the next section *

- ○ Relevant
- ○ Not relevant

22. Please provide the name of the species/community/habitat/ecosystem/locality (e.g. reserve)/bioregion (e.g. Lowveld) you are referring to for the above question *

23. Did lockdown restrictions have any influence on the impacts of ENERGY PRODUCTION AND MINING on the species/community/habitat/ecosystem/locality (e.g. reserve)/bioregion (e.g. Lowveld) for which you have expertise? *

- ○ Yes
- ○ Suspected/likely
- ○ No
- ○ Unsure

24. How did the lockdown restrictions influence the above threat to the species/community /habitat/ecosystem/locality (e.g. reserve)/bioregion (e.g. Lowveld) for which you have expertise? *

	Threat reduced	Threat increased	Threat stable	Impact on threat suspected but direction unknown	New threat
Lockdown levels 3 to 5	◯	◯	◯	◯	◯
Lockdown levels 1 to 2	◯	◯	◯	◯	◯

25. How long do you expect the effects of the lockdown restrictions on the above threat to last? *

	Short-term (for the duration of the State of Disaster)	Medium-term (3-5 years)	Long-term (>5 years)	Unknown	N/A
Lockdown levels 3 to 5	◯	◯	◯	◯	◯
Lockdown levels 1 to 2	◯	◯	◯	◯	◯

26. What was the scope of the above threat for the species/community/habitat/ecosystem /locality (e.g. reserve)/bioregion (e.g. Lowveld) for which you have expertise IMMEDIATELY BEFORE LOCKDOWN? *

	Affects the minority of the population/ community/ habitat/ ecosystem, etc. (<50%)	Affects the majority of the population/ community/ habitat/ ecosystem, etc. (50-90%)	Affects the whole population/ community/ habitat/ ecosystem, etc. (>90%)	Unknown
Immediately before lockdown	◯	◯	◯	◯

27. What is the scope of the above threat as a result of lockdown restrictions for the species/community/habitat/ecosystem/locality (e.g. reserve)/bioregion (e.g. Lowveld) for which you have expertise? *

	Affects the minority of the population/ community/ habitat/ ecosystem, etc. (<50%)	Affects the majority of the population/ community/ habitat/ ecosystem, etc. (50-90%)	Affects the whole population/ community/ habitat/ ecosystem, etc. (>90%)	Unknown
Lockdown levels 3 to 5	○	○	○	○
Lockdown levels 1 to 2	○	○	○	○

28. Please provide details of the effects you have reported above, including any data or evidence you have access to (indicate "N/A" if none) *

The impacts of TRANSPORTATION AND SERVICE CORRIDORS

These threats are from long narrow transport corridors and the vehicles that use them, including associated wildlife mortality, including but not limited to:
Roads and railroads;
Utilities and service lines (e.g. energy infrastructures);
Shipping lanes;
Flight paths

29. Is this threat relevant to your area of expertise? If not, please tick "Not relevant" to go to the next section *

- ◯ Relevant
- ◯ Not relevant

30. Please provide the name of the species/community/habitat/ecosystem/locality (e.g. reserve)/bioregion (e.g. Lowveld) you are referring to for the above question *

31. Did lockdown restrictions have any influence on the impacts of TRANSPORTATION AND SERVICE CORRIDORS on the species/community/habitat/ecosystem/locality (e.g. reserve)/bioregion (e.g. Lowveld) for which you have expertise? *

- ◯ Yes
- ◯ Suspected/likely
- ◯ No
- ◯ Unsure

32. How did the lockdown restrictions influence the above threat to the species/community /habitat/ecosystem/locality (e.g. reserve)/bioregion (e.g. Lowveld) for which you have expertise? *

	Threat reduced	Threat increased	Threat stable	Impact on threat suspected but direction unknown	New threat
Lockdown levels 3 to 5	○	○	○	○	○
Lockdown levels 1 to 2	○	○	○	○	○

33. How long do you expect the effects of the lockdown restrictions on the above threat to last? *

	Short-term (for the duration of the State of Disaster)	Medium-term (3-5 years)	Long-term (>5 years)	Unknown	N/A
Lockdown levels 3 to 5	○	○	○	○	○
Lockdown levels 1 to 2	○	○	○	○	○

34. What was the scope of the above threat for the species/community/habitat/ecosystem /locality (e.g. reserve)/bioregion (e.g. Lowveld) for which you have expertise IMMEDIATELY BEFORE LOCKDOWN? *

	Affects the minority of the population/ community/ habitat/ ecosystem, etc. (<50%)	Affects the majority of the population/ community/ habitat/ ecosystem, etc. (50-90%)	Affects the whole population/ community/ habitat/ ecosystem, etc. (>90%)	Unknown
Immediately before lockdown	○	○	○	○

35. What is the scope of the above threat as a result of lockdown restrictions for the species/community/habitat/ecosystem/locality (e.g. reserve)/bioregion (e.g. Lowveld) for which you have expertise? *

	Affects the minority of the population/ community/ habitat/ ecosystem, etc. (<50%)	Affects the majority of the population/ community/ habitat/ ecosystem, etc. (50-90%)	Affects the whole population/ community/ habitat/ ecosystem, etc. (>90%)	Unknown
Lockdown levels 3 to 5	◯	◯	◯	◯
Lockdown levels 1 to 2	◯	◯	◯	◯

36. Please provide details of the effects you have reported above, including any data or evidence you have access to (indicate "N/A" if none) *

The impacts of BIOLOGICAL RESOURCE USE

These threats are from consumptive use of "wild" biological resources including both deliberate and unintentional harvesting effects; also persecution or control of specific species, including but not limited to:
Hunting & Collecting Terrestrial Animals;
Gathering Terrestrial Plants;
Logging & Wood Harvesting;
Fishing & Harvesting Aquatic Resources

37. Is this threat relevant to your area of expertise? If not, please tick "Not relevant" to go to the next section *

- ◯ Relevant
- ◯ Not relevant

38. Please provide the name of the species/community/habitat/ecosystem/locality (e.g. reserve)/bioregion (e.g. Lowveld) you are referring to for the above question *

39. Did lockdown restrictions have any influence on the impacts of BIOLOGICAL RESOURCE USE on the species/community/habitat/ecosystem/locality (e.g. reserve)/bioregion (e.g. Lowveld) for which you have expertise? *

- ◯ Yes
- ◯ Suspected/likely
- ◯ No
- ◯ Unsure

40. How did the lockdown restrictions influence the above threat to the species/community/habitat/ecosystem/locality (e.g. reserve)/bioregion (e.g. Lowveld) for which you have expertise? *

	Threat reduced	Threat increased	Threat stable	Impact on threat suspected but direction unknown	New threat
Lockdown levels 3 to 5	○	○	○	○	○
Lockdown levels 1 to 2	○	○	○	○	○

41. How long do you expect the effects of the lockdown restrictions on the above threat to last? *

	Short-term (for the duration of the State of Disaster)	Medium-term (3-5 years)	Long-term (>5 years)	Unknown	N/A
Lockdown levels 3 to 5	○	○	○	○	○
Lockdown levels 1 to 2	○	○	○	○	○

42. What was the scope of the above threat for the species/community/habitat/ecosystem/locality (e.g. reserve)/bioregion (e.g. Lowveld) for which you have expertise IMMEDIATELY BEFORE LOCKDOWN? *

	Affects the minority of the population/ community/ habitat/ ecosystem, etc. (<50%)	Affects the majority of the population/ community/ habitat/ ecosystem, etc. (50-90%)	Affects the whole population/ community/ habitat/ ecosystem, etc. (>90%)	Unknown
Immediately before lockdown	○	○	○	○

43. What is the scope of the above threat as a result of lockdown restrictions for the species/community/habitat/ecosystem/locality (e.g. reserve)/bioregion (e.g. Lowveld) for which you have expertise? *

	Affects the minority of the population/ community/ habitat/ ecosystem, etc. (<50%)	Affects the majority of the population/ community/ habitat/ ecosystem, etc. (50-90%)	Affects the whole population/ community/ habitat/ ecosystem, etc. (>90%)	Unknown
Lockdown levels 3 to 5	○	○	○	○
Lockdown levels 1 to 2	○	○	○	○

44. Please provide details of the effects you have reported above, including any data or evidence you have access to (indicate "N/A" if none) *

The impacts of HUMAN INTRUSIONS AND DISTURBANCE

These threats are from human activities that alter, destroy and disturb habitats and species associated with non-consumptive uses of biological resources, including but not limited to:
Recreational activities;
War, Civil Unrest & Military Exercises;
Work & Other Activities (i.e. people spending time in or traveling in natural environments for reasons other than recreation or military activities), legal or illegal

45. Is this threat relevant to your area of expertise? If not, please tick "Not relevant" to go to the next section *

- ◯ Relevant
- ◯ Not relevant

46. Please provide the name of the species/community/habitat/ecosystem/locality (e.g. reserve)/bioregion (e.g. Lowveld) you are referring to for the above question *

47. Did lockdown restrictions have any influence on the impacts of HUMAN INTRUSIONS AND DISTURBANCE on the species/community/habitat/ecosystem/locality (e.g. reserve)/bioregion (e.g. Lowveld) for which you have expertise? *

- ◯ Yes
- ◯ Suspected/likely
- ◯ No
- ◯ Unsure

48. How did the lockdown restrictions influence the above threat to the species/community /habitat/ecosystem/locality (e.g. reserve)/bioregion (e.g. Lowveld) for which you have expertise? *

	Threat reduced	Threat increased	Threat stable	Impact on threat suspected but direction unknown	New threat
Lockdown levels 3 to 5	◯	◯	◯	◯	◯
Lockdown levels 1 to 2	◯	◯	◯	◯	◯

49. How long do you expect the effects of the lockdown restrictions on the above threat to last? *

	Short-term (for the duration of the State of Disaster)	Medium-term (3-5 years)	Long-term (>5 years)	Unknown	N/A
Lockdown levels 3 to 5	◯	◯	◯	◯	◯
Lockdown levels 1 to 2	◯	◯	◯	◯	◯

50. What was the scope of the above threat for the species/community/habitat/ecosystem /locality (e.g. reserve)/bioregion (e.g. Lowveld) for which you have expertise IMMEDIATELY BEFORE LOCKDOWN? *

	Affects the minority of the population/ community/ habitat/ ecosystem, etc. (<50%)	Affects the majority of the population/ community/ habitat/ ecosystem, etc. (50-90%)	Affects the whole population/ community/ habitat/ ecosystem, etc. (>90%)	Unknown
Immediately before lockdown	◯	◯	◯	◯

51. What is the scope of the above threat as a result of lockdown restrictions for the species/community/habitat/ecosystem/locality (e.g. reserve)/bioregion (e.g. Lowveld) for which you have expertise? *

	Affects the minority of the population/ community/ habitat/ ecosystem, etc. (<50%)	Affects the majority of the population/ community/ habitat/ ecosystem, etc. (50-90%)	Affects the whole population/ community/ habitat/ ecosystem, etc. (>90%)	Unknown
Lockdown levels 3 to 5	○	○	○	○
Lockdown levels 1 to 2	○	○	○	○

52. Please provide details of the effects you have reported above, including any data or evidence you have access to (indicate "N/A" if none) *

The impacts of NATURAL SYSTEM MODIFICATION

These threats are from actions that convert or degrade habitat in service of "managing" natural or semi-natural systems, often to improve human welfare, including but not limited to:
Fire & Fire Suppression;
Dams & Water Management/Use;
Other Ecosystem Modifications (e.g. land reclamation projects, abandonment of managed lands, mowing grass, tree thinning in parks, beach construction, removal of snags from streams, etc.) legal or illegal

53. Is this threat relevant to your area of expertise? If not, please tick "Not relevant" to go to the next section *

- ◯ Relevant
- ◯ Not relevant

54. Please provide the name of the species/community/habitat/ecosystem/locality (e.g. reserve)/bioregion (e.g. Lowveld) you are referring to for the above question *

55. Did lockdown restrictions have any influence on the impacts of NATURAL SYSTEM MODIFICATION on the species/community/habitat/ecosystem/locality (e.g. reserve)/bioregion (e.g. Lowveld) for which you have expertise? *

- ◯ Yes
- ◯ Suspected/likely
- ◯ No
- ◯ Unsure

56. How did the lockdown restrictions influence the above threat to the species/community /habitat/ecosystem/locality (e.g. reserve)/bioregion (e.g. Lowveld) for which you have expertise? *

	Threat reduced	Threat increased	Threat stable	Impact on threat suspected but direction unknown	New threat
Lockdown levels 3 to 5	◯	◯	◯	◯	◯
Lockdown levels 1 to 2	◯	◯	◯	◯	◯

57. How long do you expect the effects of the lockdown restrictions on the above threat to last? *

	Short-term (for the duration of the State of Disaster)	Medium-term (3-5 years)	Long-term (>5 years)	Unknown	N/A
Lockdown levels 3 to 5	◯	◯	◯	◯	◯
Lockdown levels 1 to 2	◯	◯	◯	◯	◯

58. What was the scope of the above threat for the species/community/habitat/ecosystem /locality (e.g. reserve)/bioregion (e.g. Lowveld) for which you have expertise IMMEDIATELY BEFORE LOCKDOWN? *

	Affects the minority of the population/ community/ habitat/ ecosystem, etc. (<50%)	Affects the majority of the population/ community/ habitat/ ecosystem, etc. (50-90%)	Affects the whole population/ community/ habitat/ ecosystem, etc. (>90%)	Unknown
Immediately before lockdown	◯	◯	◯	◯

59. What is the scope of the above threat as a result of lockdown restrictions for the species/community/habitat/ecosystem/locality (e.g. reserve)/bioregion (e.g. Lowveld) for which you have expertise? *

	Affects the minority of the population/ community/ habitat/ ecosystem, etc. (<50%)	Affects the majority of the population/ community/ habitat/ ecosystem, etc. (50-90%)	Affects the whole population/ community/ habitat/ ecosystem, etc. (>90%)	Unknown
Lockdown levels 3 to 5	○	○	○	○
Lockdown levels 1 to 2	○	○	○	○

60. Please provide details of the effects you have reported above, including any data or evidence you have access to (indicate "N/A" if none) *

Impacts of INVASIVE AND OTHER PROBLEMATIC SPECIES, GENES AND DISEASES

These threats are from non-native and native plants, animals, pathogens/microbes, or genetic materials that have or are predicted to have harmful effects on biodiversity following their introduction, spread and/or increase in abundance, including but not limited to:
Invasive Non-Native/Alien Species/Diseases;
Problematic Native Species/Diseases;
Introduced Genetic Material;
Problematic Species/Diseases of Unknown Origin;
Viral/Prion-induced Diseases;
Diseases of Unknown Cause

61. Is this threat relevant to your area of expertise? If not, please tick "Not relevant" to go to the next section *

- ◯ Relevant
- ◯ Not relevant

62. Please provide the name of the species/community/habitat/ecosystem/locality (e.g. reserve)/bioregion (e.g. Lowveld) you are referring to for the above question *

63. Did lockdown restrictions have any influence on the impacts of INVASIVE AND OTHER PROBLEMATIC SPECIES, GENES AND DISEASES on the species/community/habitat /ecosystem/locality (e.g. reserve)/bioregion (e.g. Lowveld) for which you have expertise? *

- ◯ Yes
- ◯ Suspected/likely
- ◯ No
- ◯ Unsure

64. How did the lockdown restrictions influence the above threat to the species/community /habitat/ecosystem/locality (e.g. reserve)/bioregion (e.g. Lowveld) for which you have expertise? *

	Threat reduced	Threat increased	Threat stable	Impact on threat suspected but direction unknown	New threat
Lockdown levels 3 to 5	◯	◯	◯	◯	◯
Lockdown levels 1 to 2	◯	◯	◯	◯	◯

65. How long do you expect the effects of the lockdown restrictions on the above threat to last? *

	Short-term (for the duration of the State of Disaster)	Medium-term (3-5 years)	Long-term (>5 years)	Unknown	N/A
Lockdown levels 3 to 5	◯	◯	◯	◯	◯
Lockdown levels 1 to 2	◯	◯	◯	◯	◯

66. What was the scope of the above threat for the species/community/habitat/ecosystem /locality (e.g. reserve)/bioregion (e.g. Lowveld) for which you have expertise IMMEDIATELY BEFORE LOCKDOWN? *

	Affects the minority of the population/ community/ habitat/ ecosystem, etc. (<50%)	Affects the majority of the population/ community/ habitat/ ecosystem, etc. (50-90%)	Affects the whole population/ community/ habitat/ ecosystem, etc. (>90%)	Unknown
Immediately before lockdown	○	○	○	○

67. What is the scope of the above threat as a result of lockdown restrictions for the species/community/habitat/ecosystem/locality (e.g. reserve)/bioregion (e.g. Lowveld) for which you have expertise? *

	Affects the minority of the population/ community/ habitat/ ecosystem, etc. (<50%)	Affects the majority of the population/ community/ habitat/ ecosystem, etc. (50-90%)	Affects the whole population/ community/ habitat/ ecosystem, etc. (>90%)	Unknown
Lockdown levels 3 to 5	○	○	○	○
Lockdown levels 1 to 2	○	○	○	○

68. Please provide details of the effects you have reported above, including any data or evidence you have access to (indicate "N/A" if none) *

The impacts of POLLUTION

These threats are from the introduction of exotic and/or excess materials or energy from point and nonpoint sources, including but not limited to:
Domestic & Urban Waste Water;
Industrial & Military Effluents;
Agricultural & Forestry Effluents;
Garbage & Solid Waste;
Air-Borne Pollutants;
Excess Energy (e.g. light, thermal, noise pollution)

69. Is this threat relevant to your area of expertise? If not, please tick "Not relevant" to go to the next section *

- ◯ Relevant
- ◯ Not relevant

70. Please provide the name of the species/community/habitat/ecosystem/locality (e.g. reserve)/bioregion (e.g. Lowveld) you are referring to for the above question *

71. Did lockdown restrictions have any influence on the impacts of POLLUTION on the species/community/habitat/ecosystem/locality (e.g. reserve)/bioregion (e.g. Lowveld) for which you have expertise? *

- ◯ Yes
- ◯ Suspected/likely
- ◯ No
- ◯ Unsure

72. How did the lockdown restrictions influence the above threat to the species/community /habitat/ecosystem/locality (e.g. reserve)/bioregion (e.g. Lowveld) for which you have expertise? *

	Threat reduced	Threat increased	Threat stable	Impact on threat suspected but direction unknown	New threat
Lockdown levels 3 to 5	○	○	○	○	○
Lockdown levels 1 to 2	○	○	○	○	○

73. How long do you expect the effects of the lockdown restrictions on the above threat to last? *

	Short-term (for the duration of the State of Disaster)	Medium-term (3-5 years)	Long-term (>5 years)	Unknown	N/A
Lockdown levels 3 to 5	○	○	○	○	○
Lockdown levels 1 to 2	○	○	○	○	○

74. What was the scope of the above threat for the species/community/habitat/ecosystem /locality (e.g. reserve)/bioregion (e.g. Lowveld) for which you have expertise IMMEDIATELY BEFORE LOCKDOWN? *

	Affects the minority of the population/ community/ habitat/ ecosystem, etc. (<50%)	Affects the majority of the population/ community/ habitat/ ecosystem, etc. (50-90%)	Affects the whole population/ community/ habitat/ ecosystem, etc. (>90%)	Unknown
Immediately before lockdown	○	○	○	○

75. What is the scope of the above threat as a result of lockdown restrictions for the species/community/habitat/ecosystem/locality (e.g. reserve)/bioregion (e.g. Lowveld) for which you have expertise? *

	Affects the minority of the population/ community/ habitat/ ecosystem, etc. (<50%)	Affects the majority of the population/ community/ habitat/ ecosystem, etc. (50-90%)	Affects the whole population/ community/ habitat/ ecosystem, etc. (>90%)	Unknown
Lockdown levels 3 to 5	○	○	○	○
Lockdown levels 1 to 2	○	○	○	○

76. Please provide details of the effects you have reported above, including any data or evidence you have access to (indicate "N/A" if none) *

The impacts of CLIMATE CHANGE AND SEVERE WEATHER

These threats are from long-term climatic changes which may be linked to global warming and other severe climatic/weather events that are outside of the natural range of variation, or potentially can wipe out a vulnerable species or habitat, including but not limited to:
Habitat Shifting & Alteration;
Droughts;
Temperature Extremes;
Storms & Flooding;
Other Impacts

77. Is this threat relevant to your area of expertise? If not, please tick "Not relevant" to go to the next section *

◯ Relevant

◯ Not relevant

78. Please provide the name of the species/community/habitat/ecosystem/locality (e.g. reserve)/bioregion (e.g. Lowveld) you are referring to for the above question *

79. Did lockdown restrictions have any influence on the impacts of CLIMATE CHANGE AND SEVERE WEATHER on the species/community/habitat/ecosystem/locality (e.g. reserve)/bioregion (e.g. Lowveld) for which you have expertise? *

◯ Yes

◯ Suspected/likely

◯ No

◯ Unsure

80. How did the lockdown restrictions influence the above threat to the species/community/habitat/ecosystem/locality (e.g. reserve)/bioregion (e.g. Lowveld) for which you have expertise? *

	Threat reduced	Threat increased	Threat stable	Impact on threat suspected but direction unknown	New threat
Lockdown levels 3 to 5	○	○	○	○	○
Lockdown levels 1 to 2	○	○	○	○	○

81. How long do you expect the effects of the lockdown restrictions on the above threat to last? *

	Short-term (for the duration of the State of Disaster)	Medium-term (3-5 years)	Long-term (>5 years)	Unknown	N/A
Lockdown levels 3 to 5	○	○	○	○	○
Lockdown levels 1 to 2	○	○	○	○	○

82. What was the scope of the above threat for the species/community/habitat/ecosystem/locality (e.g. reserve)/bioregion (e.g. Lowveld) for which you have expertise IMMEDIATELY BEFORE LOCKDOWN? *

	Affects the minority of the population/ community/ habitat/ ecosystem, etc. (<50%)	Affects the majority of the population/ community/ habitat/ ecosystem, etc. (50-90%)	Affects the whole population/ community/ habitat/ ecosystem, etc. (>90%)	Unknown
Immediately before lockdown	○	○	○	○

83. What is the scope of the above threat as a result of lockdown restrictions for the species/community/habitat/ecosystem/locality (e.g. reserve)/bioregion (e.g. Lowveld) for which you have expertise? *

	Affects the minority of the population/ community/ habitat/ ecosystem, etc. (<50%)	Affects the majority of the population/ community/ habitat/ ecosystem, etc. (50-90%)	Affects the whole population/ community/ habitat/ ecosystem, etc. (>90%)	Unknown
Lockdown levels 3 to 5	◯	◯	◯	◯
Lockdown levels 1 to 2	◯	◯	◯	◯

84. Please provide details of the effects you have reported above, including any data or evidence you have access to (indicate "N/A" if none) *

85. Please add any impacts that Covid-19 lockdown restrictions have had on your normal ability to conduct conservation activities/research/monitoring/education/outreach (indicate "N/A" if none) *

Further impacts not covered by the IUCN Taxonomy of Threats

86. Please add any further quantifiable impacts of the Covid-19 pandemic/lockdown not covered
e.g. loss of funding for conservation/research; loss of human capital in terms of conservation researchers, etc. (indicate "N/A" if none) *

This content is neither created nor endorsed by Microsoft. The data you submit will be sent to the form owner.

Microsoft Forms

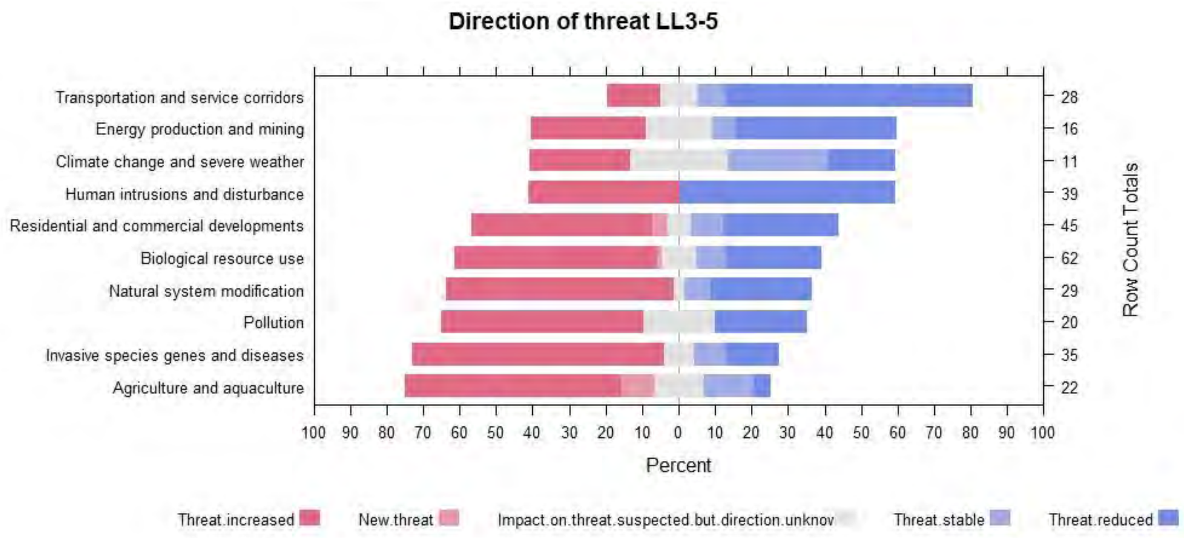

Supplementary information Figure S1a Direction of threats during levels 3-5 of Covid-19 lockdown restrictions in South Africa

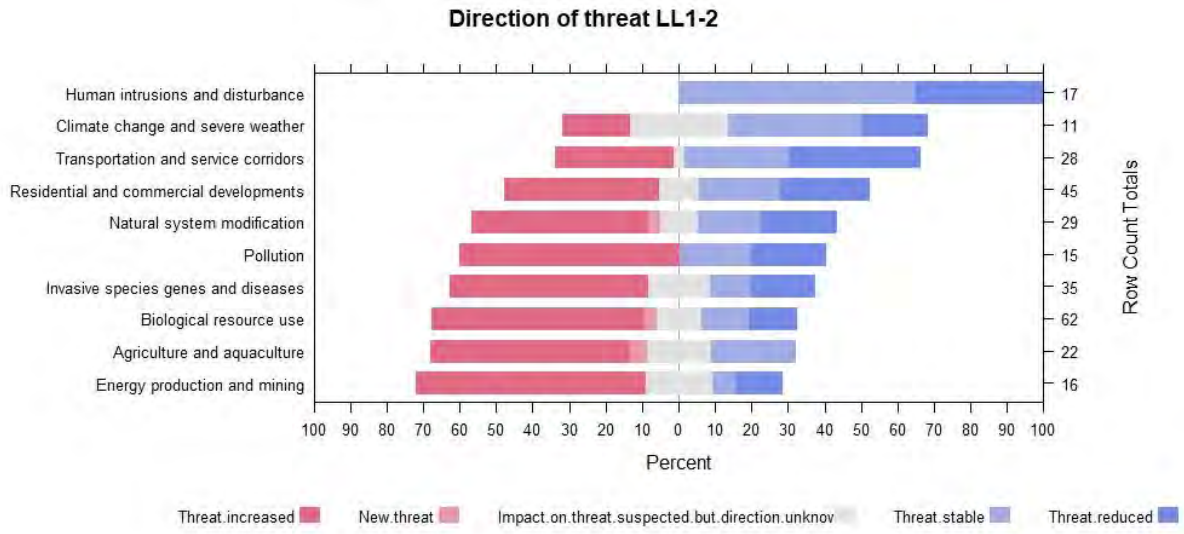

Supplementary information Figure S1b Direction of threats during levels 1-2 of Covid-19 lockdown restrictions in South Africa

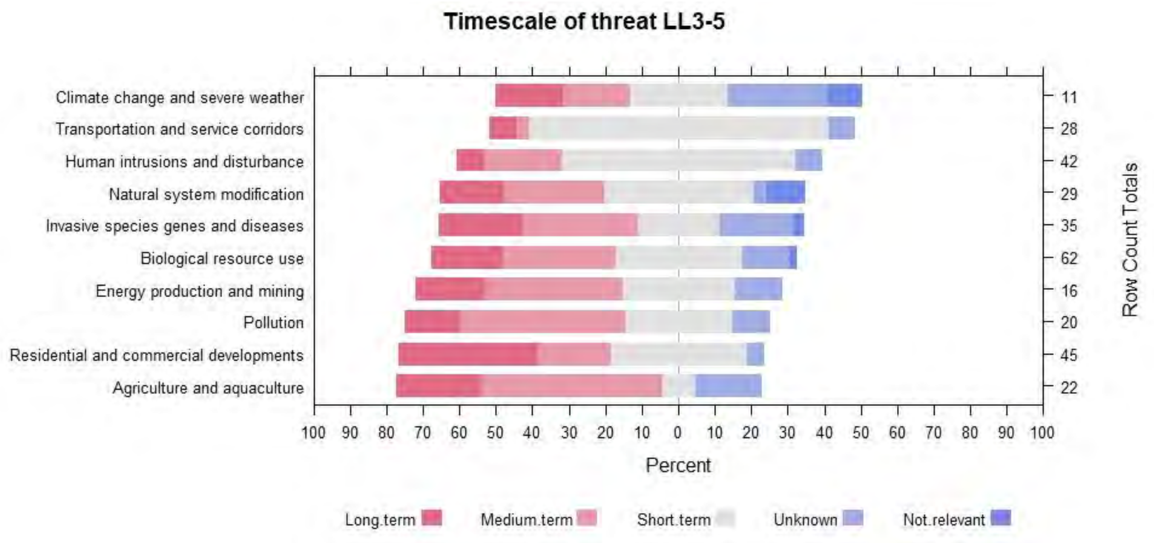

Supplementary information Figure S1c Timescale of threats during levels 3-5 of Covid-19 lockdown restrictions in South Africa

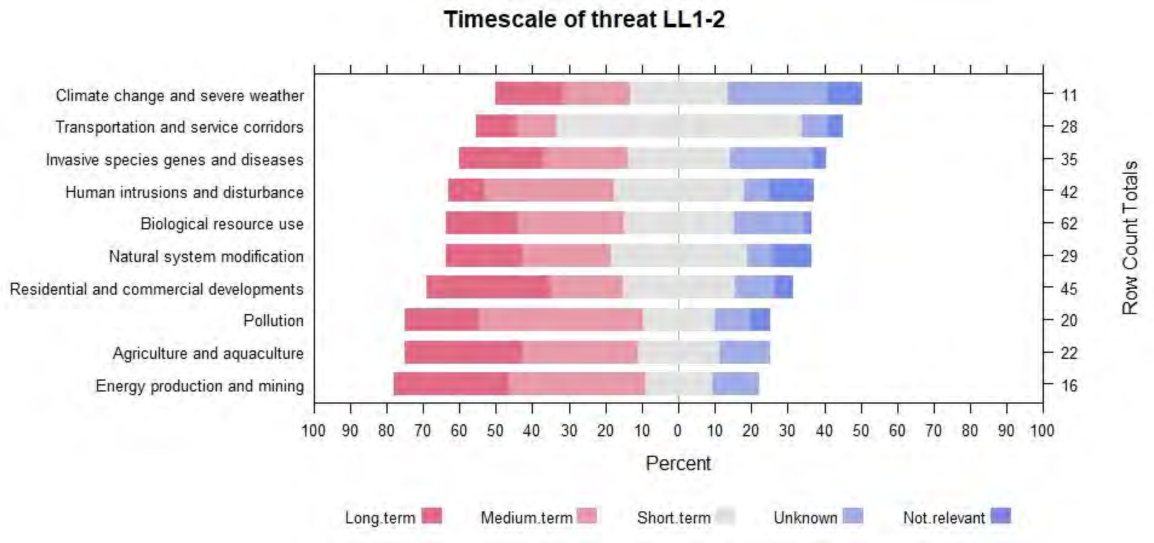

Supplementary information Figure S1d Timescale of threats during levels 1-2 of Covid-19 lockdown restrictions in South Africa

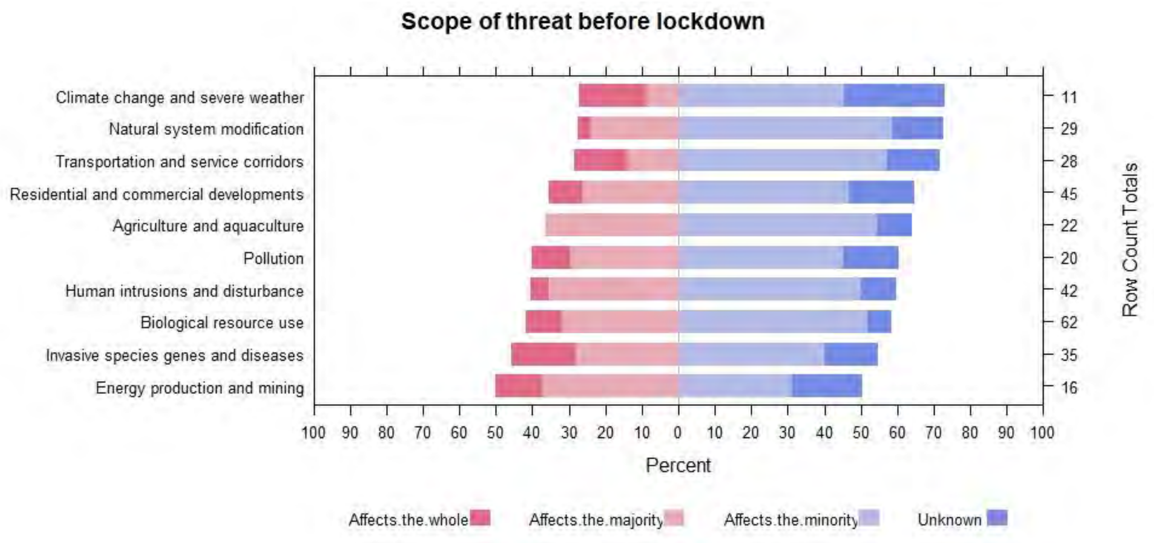

Supplementary information Figure S1e Scope of threats immediately before Covid-19 lockdown restrictions in South Africa

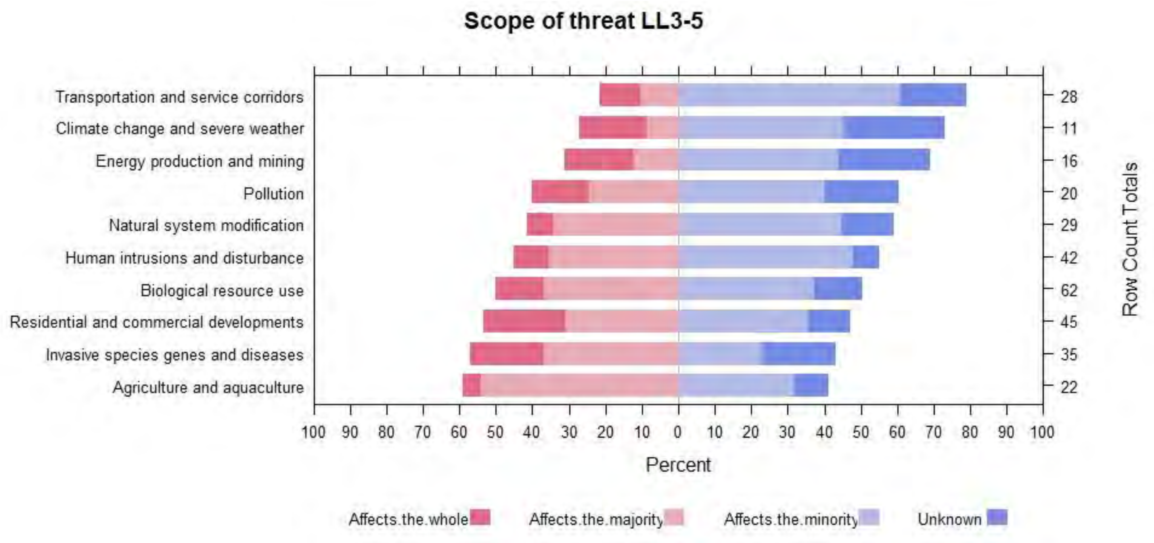

Supplementary information Figure S1f Scope of threats during levels 3-5 of Covid-19 lockdown restrictions in South Africa

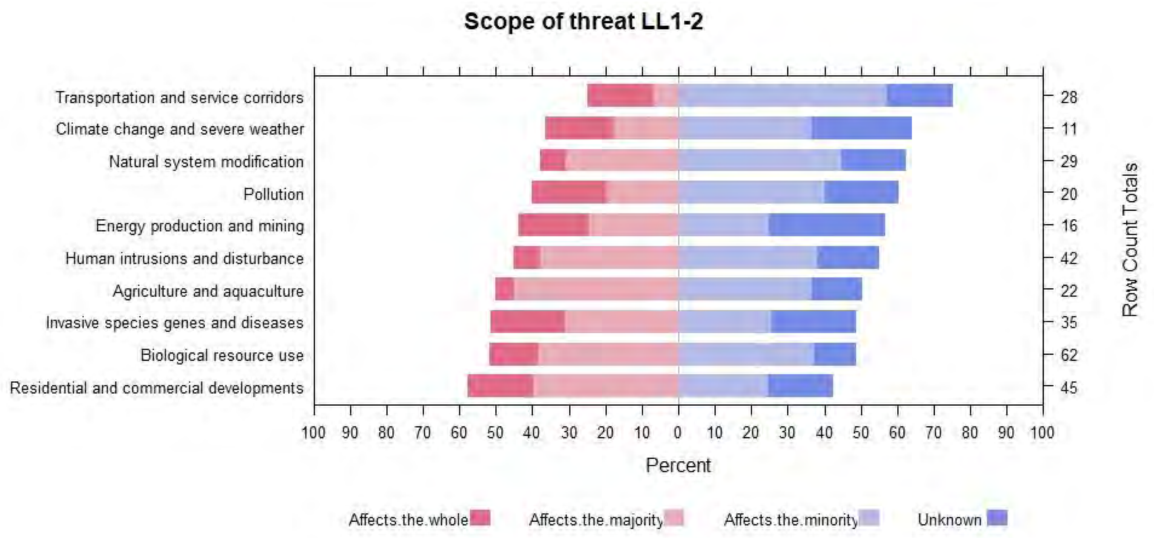

Supplementary information Figure S1g Scope of threats during levels 1-2 of Covid-19 lockdown restrictions in South Africa
